# Supplementary material for: Conserved function of the HAUS6 calponin homology domain in anchoring augmin for microtubule branching
Source: Nat Commun. 2025 Aug 22;16:7845. doi: 10.1038/s41467-025-63165-z (PMC12373997; doi:10.1038/s41467-025-63165-z)
Supplement: Supplementary file 1 — Supplementary Information [file 41467_2025_63165_MOESM1_ESM.pdf]

Supplementary Information for

Conserved function of the HAUS6 calponin homology domain in anchoring augmin for microtubule branching

Martin Würtz [1,2,*,#], Giulia Tonon[1,3,*], Bram J.A. Vermeulen[1], Maja Zezlina[1], Qi Gao[1], Annett Neuner[1], Angelika Seidl[4], Melanie König[5], Maximilian Harkenthal[1], Sebastian Eustermann[2], Sylvia Erhardt[4,6], Fabio Lolicato[5,7], Elmar Schiebel[1] and Stefan Pfeffer [1,#]

[1] Zentrum für Molekulare Biologie der Universität Heidelberg (ZMBH), 69120 Heidelberg, Germany

[2] European Molecular Biology Laboratory (EMBL), 69117 Heidelberg, Germany

[3] Current address: Department of Molecular Sociology, Max Planck Institute of Biophysics, 60438 Frankfurt, Germany

[4] Zoological Institute, Karlsruhe Institute of Technology (KIT), 76131 Karlsruhe, Germany

[5] Current address: Biochemie-Zentrum der Universität Heidelberg (BZH), 69120 Heidelberg, Germany

[6] Institute of Biological and Chemical Systems - Functional Molecular Systems (IBACS-FMS), Karlsruhe Institute of Technology (KIT), Germany

[7] Department of Physics, University of Helsinki, Helsinki, Finland

* These authors contributed equally

# correspondence to Martin Würtz (m.wuertz@zmbh.uni-heidelberg.de) and Stefan Pfeffer (s.pfeffer@zmbh.uni-heidelberg.de)

Supplementary Figures

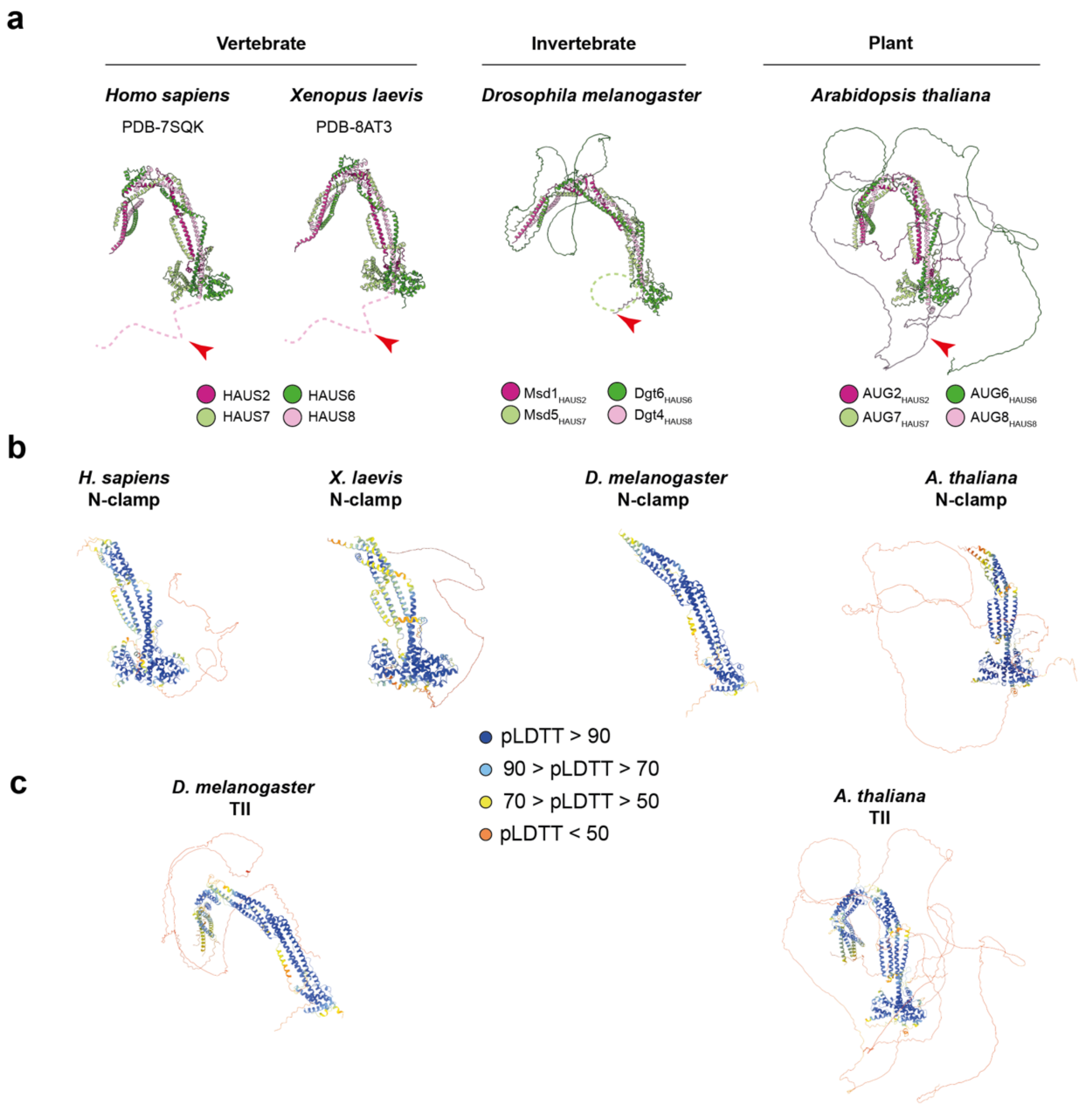

Supplementary Figure 1. AlphaFold 2 structure prediction of augmin TII. a First-ranked AlphaFold 2 (AF2) predictions (*A. thaliana*, *D. melanogaster*) or published PDB structures (human, *X. laevis*) of TII subcomplexes. Red arrowheads indicate unstructured N-termini of HAUS8 orthologs, either predicted by AF2 or indicated by dashed lines. **b** The first-ranked AF2 predictions for augmin N-clamps shown in Fig. 1c colored by pLDDT. For *H. sapiens*, *X. laevis and A. thaliana* the EGFP tag on the C-terminus of HAUS7 was included in the prediction but is not shown. Predicted Aligned Error (PAE) plots are shown in Supplementary Figure 14. **c** The first-ranked AF2 predictions for augmin TII shown in panel (a) colored by pLDDT. Predicted Aligned Error (PAE) plots are shown in Supplementary Figure 19.

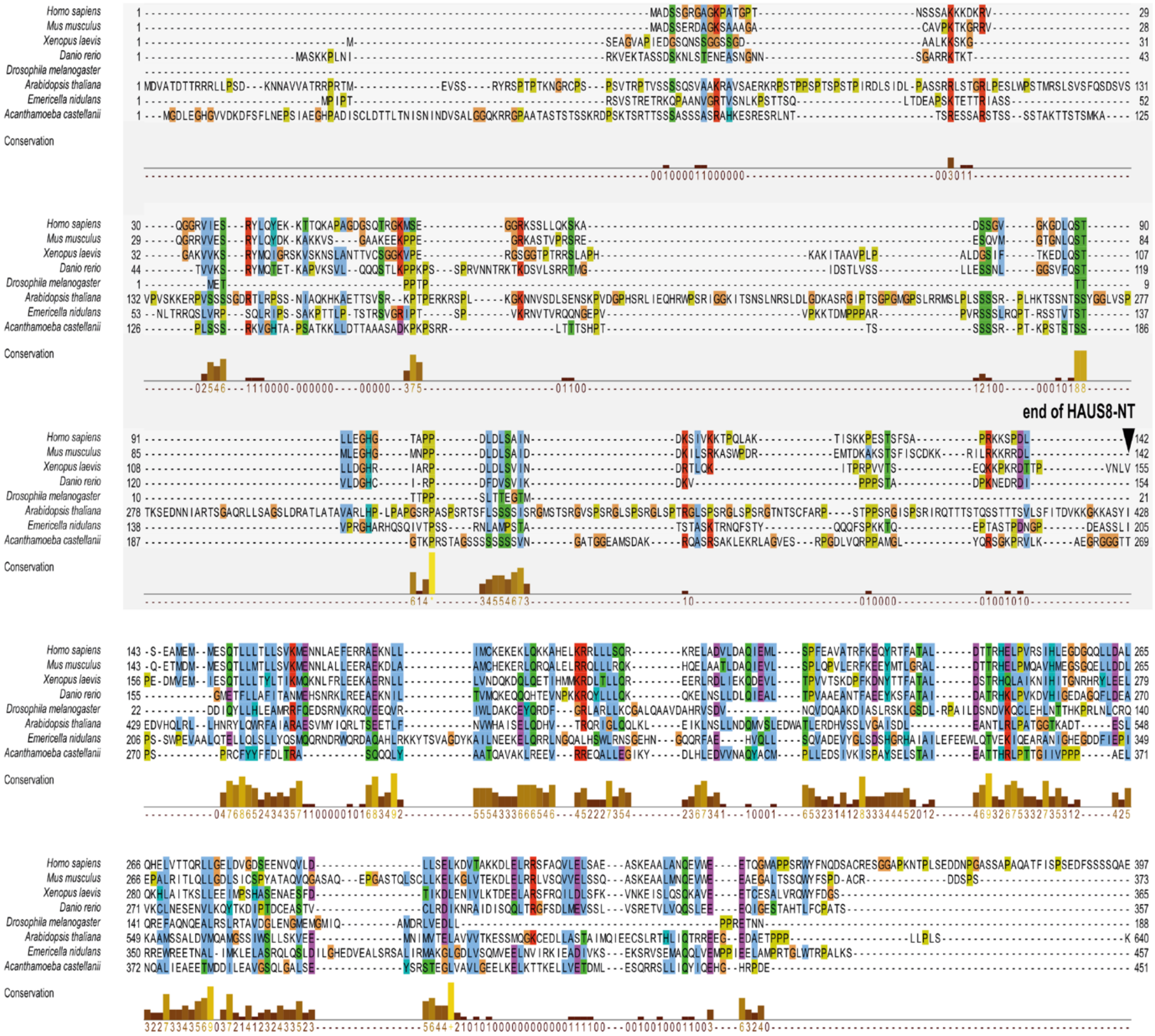

Supplementary Figure 2. Multiple sequence alignment of HAUS8 orthologs. The unstructured N-termini of HAUS8 orthologs are superposed with a grey background and the transition to structured segments of HAUS8 is indicated with black arrowhead. Multiple sequence alignment (MSA) generated with the MAFFT algorithm [1] and residues colored according to the Clustal scheme in JalView[2].

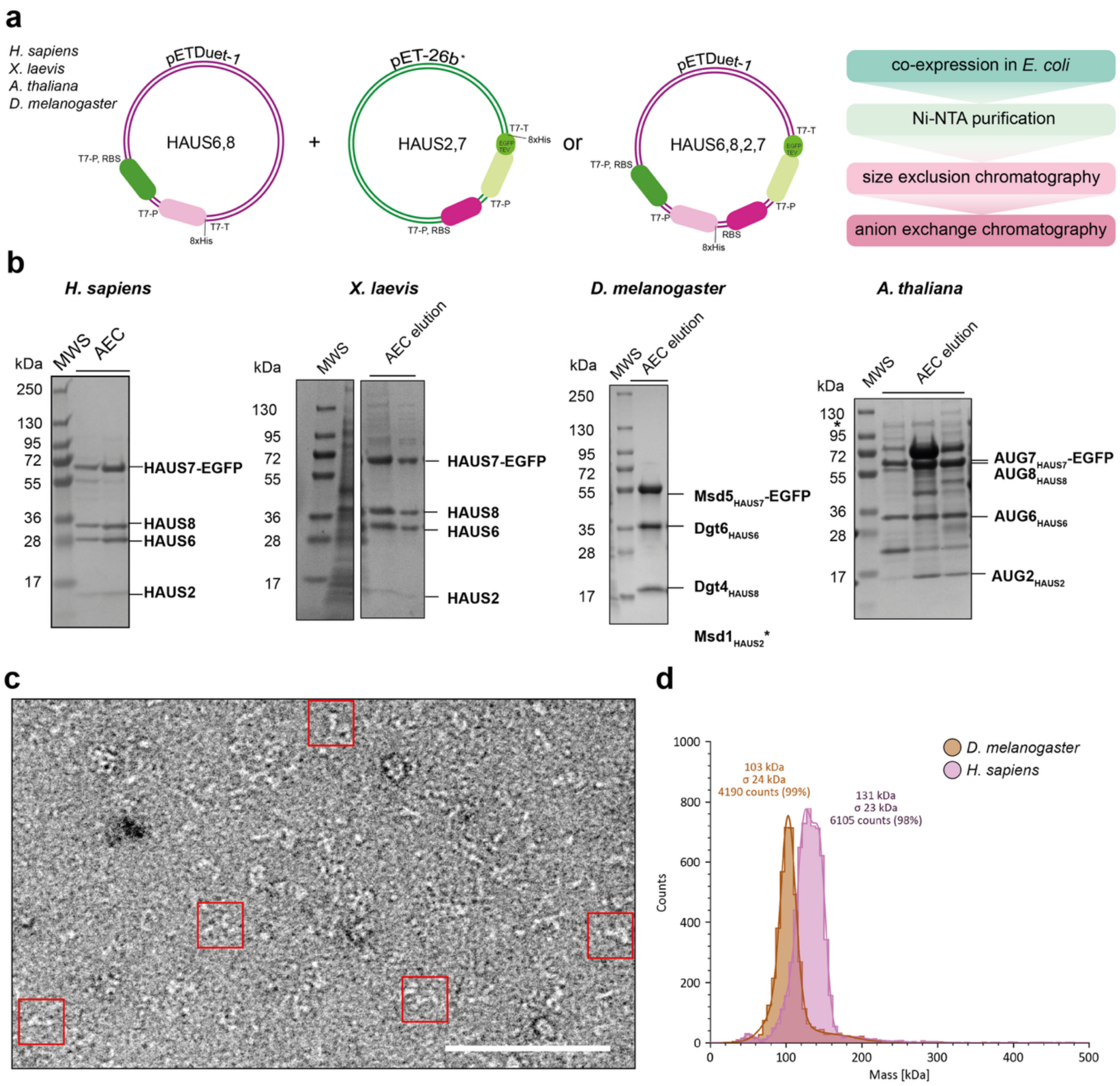

Supplementary Figure 3. Purification and characterization of augmin N-clamp variants. a Left: Expression constructs for double or single transformation into *E. coli* BL21 RIL cells. T7 promotors (T7-P), ribosome binding sites (RBS) and T7 terminator (T7-T) are indicated. Coloring of HAUS proteins: HAUS6 (dark green), HAUS8 (pink), HAUS2 (magenta), HAUS7 (light green). Right: augmin N-clamp purification strategy. **b** Section of representative Coomassie-stained SDS-PAGE gels for purified augmin N-clamps from different species. * Msd1 was not visible due to its low molecular weight. MWS: Molecular weight standards. Purifications were repeated in n=3 experiments. **c** Representative section of negative stain EM micrograph of augmin N-clamp (*H. sapiens*) after SEC with representative selected particles (red boxes). Scale bar: 100 nm. Negative stain EM was performed in n=1 experiment. **d** Histograms of mass photometry measurements for *H. sapiens* and *D. melanogaster* augmin N-clamp (n=1 experiment). Coloring as indicated. Source data are provided as a Source Data file.

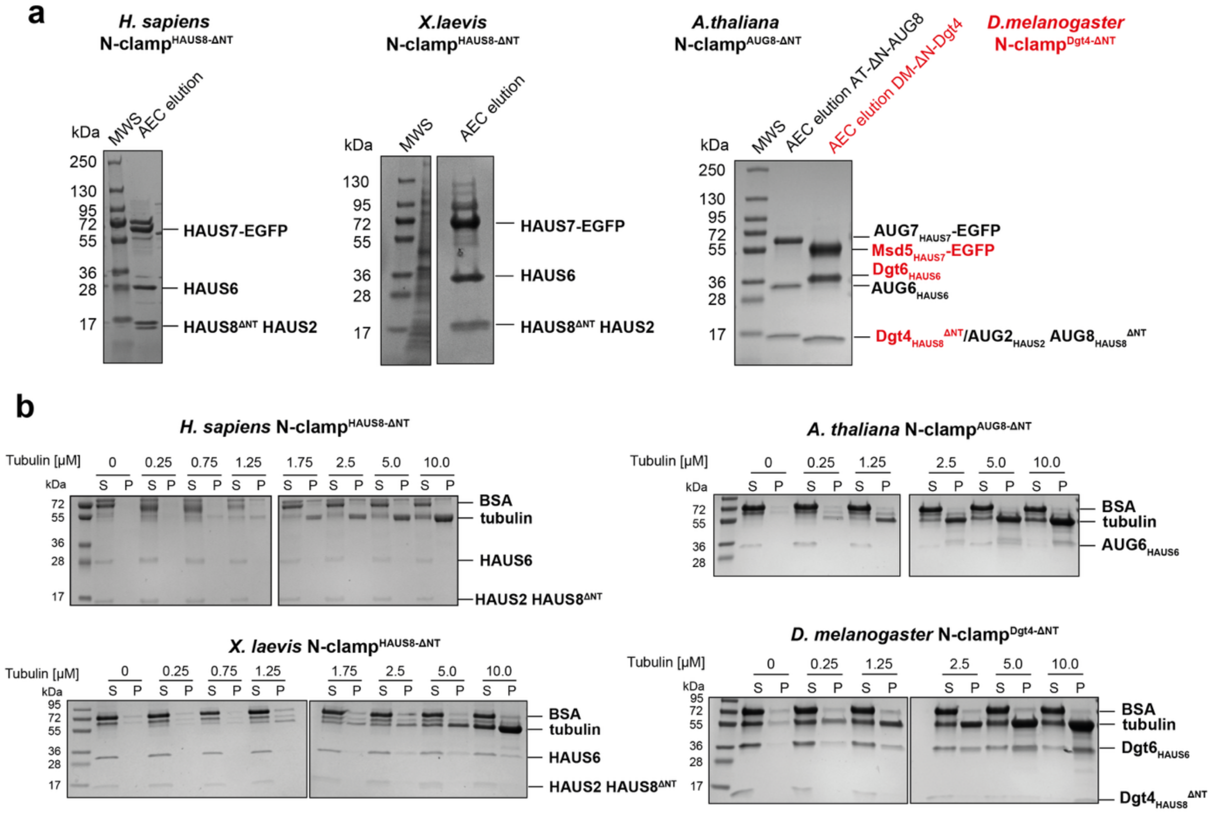

Supplementary Figure 4. Purification and MT-binding properties of HAUS8-ΔNT variants. a Sections of representative Coomassie-stained SDS-PAGE gels for purified HAUS8$^{\Delta NT}$ N-clamps from different species. *D. melanogaster* augmin N-clamp labels are shown in red. MWS: Molecular weight standards. Purifications were repeated in n=3 experiments. **b** Sections of representative SDS-PAGE gels for tubulin co-sedimentation assays of HAUS8$^{\Delta NT}$ N-clamps from different species. Tubulin concentration is indicated on the top. Supernatant (S) and Pellet (P) fractions are indicated (see Table 1). Source data are provided as a Source Data file.

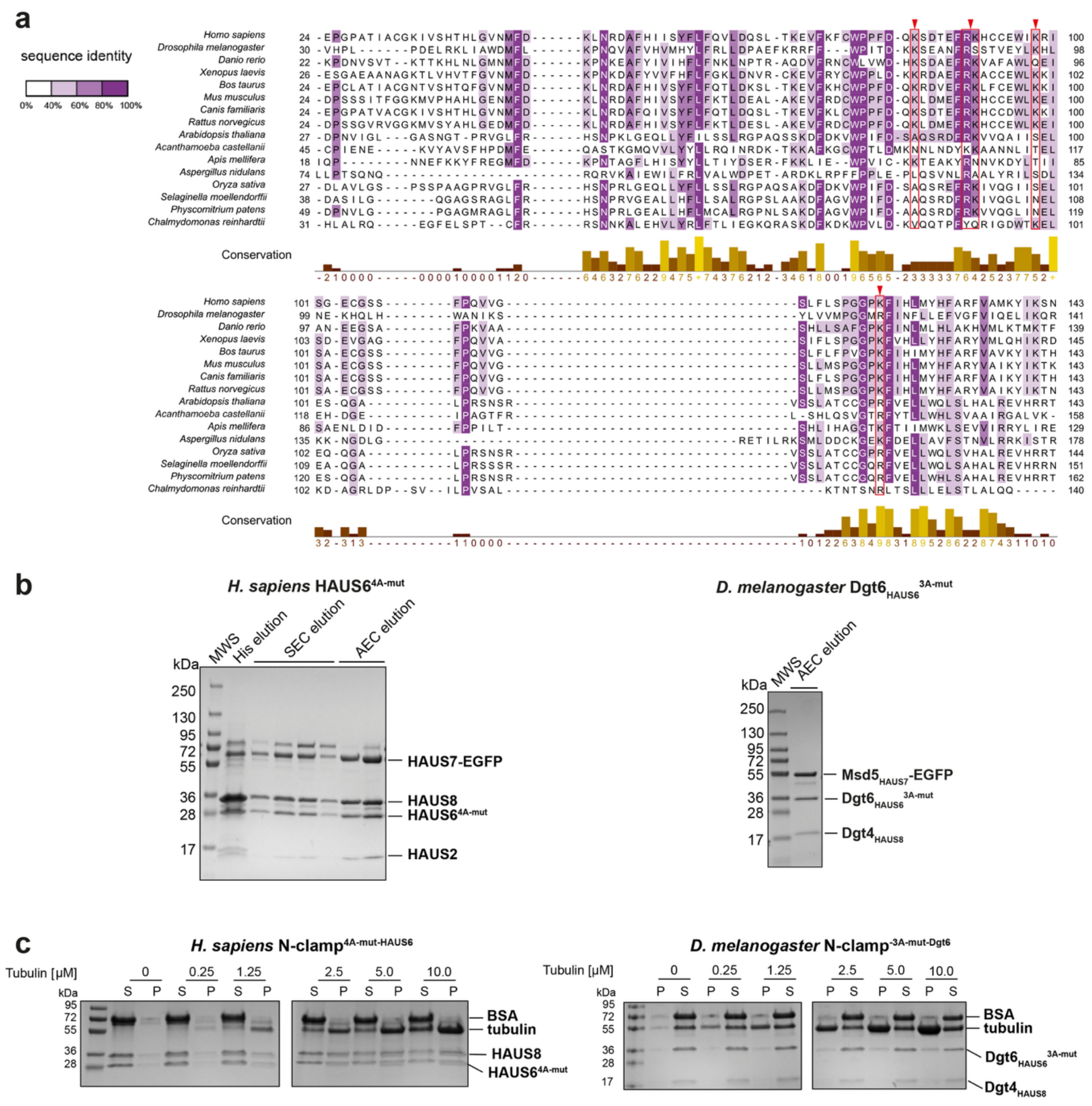

Supplementary Figure 5. Conservation of a basic patch in CH6 important for MT binding. a Multiple sequence alignment (MSA) of CH6 from various species, highlighting several conserved key residues at the potential MT-binding interface (red arrowheads). Coloring as indicated. **b** Section of representative Coomassie-stained SDS-PAGE gels for purified *H. sapiens* HAUS6$^{4A\text{-}mut}$ *(left)* and *D. melanogaster* Dgt6$_{HAUS6}^{3A\text{-}mut}$ *(right)* augmin N-clamp variants. MWS: Molecular weight standards. Purifications were repeated in n=3 experiments. **c** Sections of representative SDS-PAGE gels for tubulin co-sedimentation assays of *H. sapiens* HAUS6$^{4A\text{-}mut}$ *(right) and D. melanogaster* Dgt6$_{HAUS6}^{3A\text{-}mut}$ *(left)* N-clamp variants. Tubulin concentration is indicated on the top. Supernatant (S) and Pellet (P) fractions are indicated (see Table 1). Source data are provided as a Source Data file.

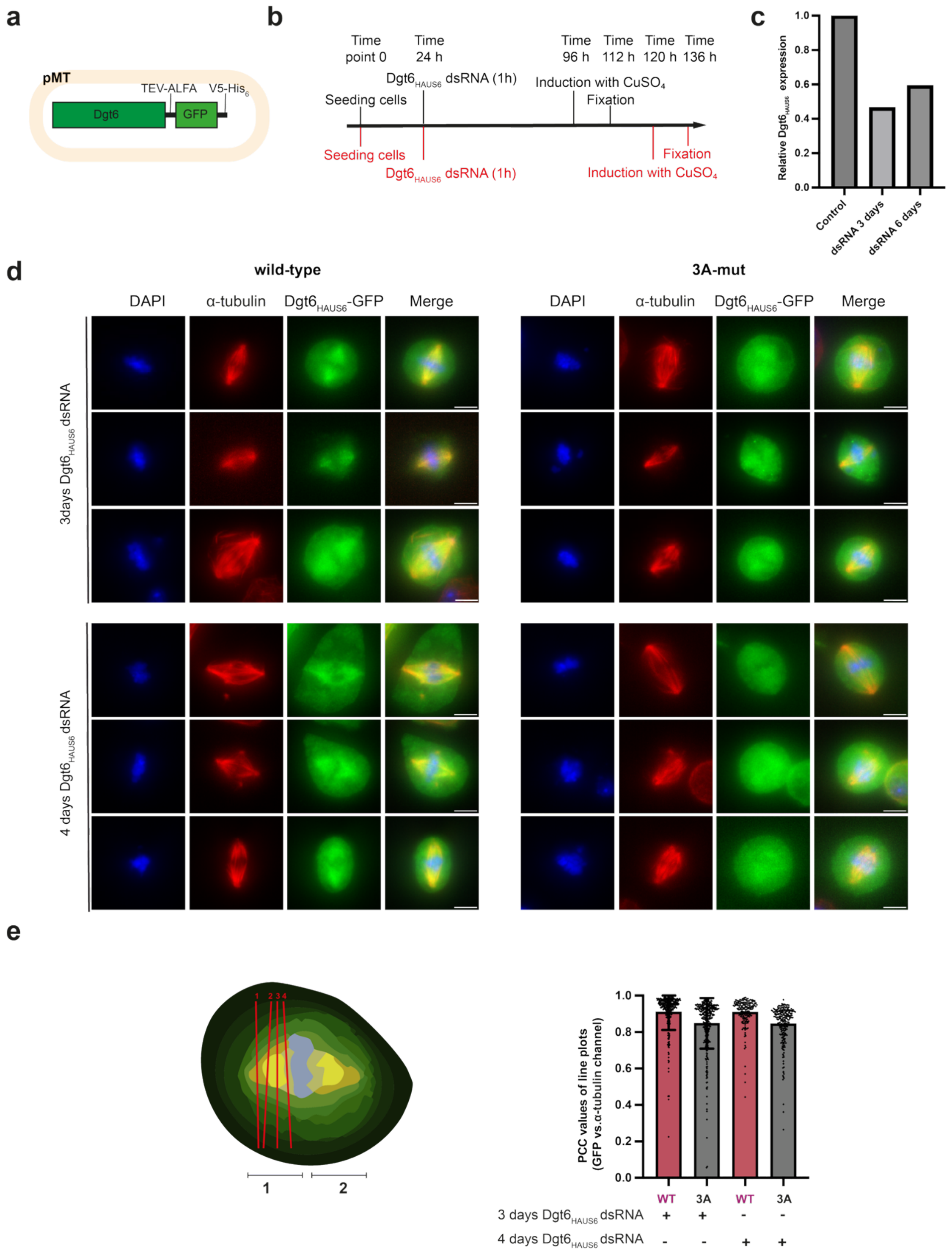

Supplementary Figure 6. CH6 is required for spindle localization in *D. melanogaster* S2 cells. a $Dgt6_{HAUS6}$-GFP constructs for expression in *D. melanogaster* S2 cells. **b** Experimental workflow for $Dgt6_{HAUS6}$ dsRNA treatment of S2 cells for either 3 days *(black, top)* or 4 days *(red, bottom)*. **c** qPCR result for $Dgt6_{HAUS6}$ dsRNA-mediated knockdown of $Dgt6_{HAUS6}$ in *Drosophila* S2 cells (shown is n=1 experiment). **d** Immunofluorescence images following $Dgt6_{HAUS6}$ dsRNA-mediated $Dgt6_{HAUS6}$ knockdown in *D. melanogaster* S2 cells. Cells expressing either $Dgt6_{HAUS6}$-GFP wild-type (WT) or 3A mut (K82A, R88A, K96A) constructs were stained for α-tubulin, DAPI, and GFP ($Dgt6_{HAUS6}$). Representative sections of images (analyzed in e) showing metaphase cells from WT *(left)* and 3A-mut *(right)* after 3 days *(top)* or 4 days *(bottom)* of $Dgt6_{HAUS6}$ dsRNA treatment. Scale bars: 5 µm. **e** Quantification of $Dgt6_{HAUS6}$ spindle localization. Left**:** Schematic representation of localization quantification. For each metaphase

spindle of $Dgt6_{HAUS6}^{WT}$-GFP or $Dgt6_{HAUS6}^{3A}$-GFP cells, four lines (red) were randomly drawn through each of the two spindle poles and the intensity values of the line plots (GFP vs α-tubulin) was compared using Pearson correlation coefficients (PCC). Right: Plot of PCC values for the two experiments after 3 days or 4 days of $Dgt6_{HAUS6}$ dsRNA treatment. The bars represent mean values and are shown in red for wild-type and gray for 3A-mut cells. Experiments of the two timepoints represent biologically independent experiments. 3 days $Dgt6_{HAUS6}$ dsRNA: WT 43 cells, 3A-mut 43 cells, n=3 experiments; 4 days $Dgt6_{HAUS6}$ dsRNA: WT 25 cells, 3A-mut 32 cells, n=1 experiments. For experiments that were repeated three times the error bars represent the SD. PCC values of both timepoints were pooled and plot is shown in Fig. 3f. Source data are provided as a Source Data file.

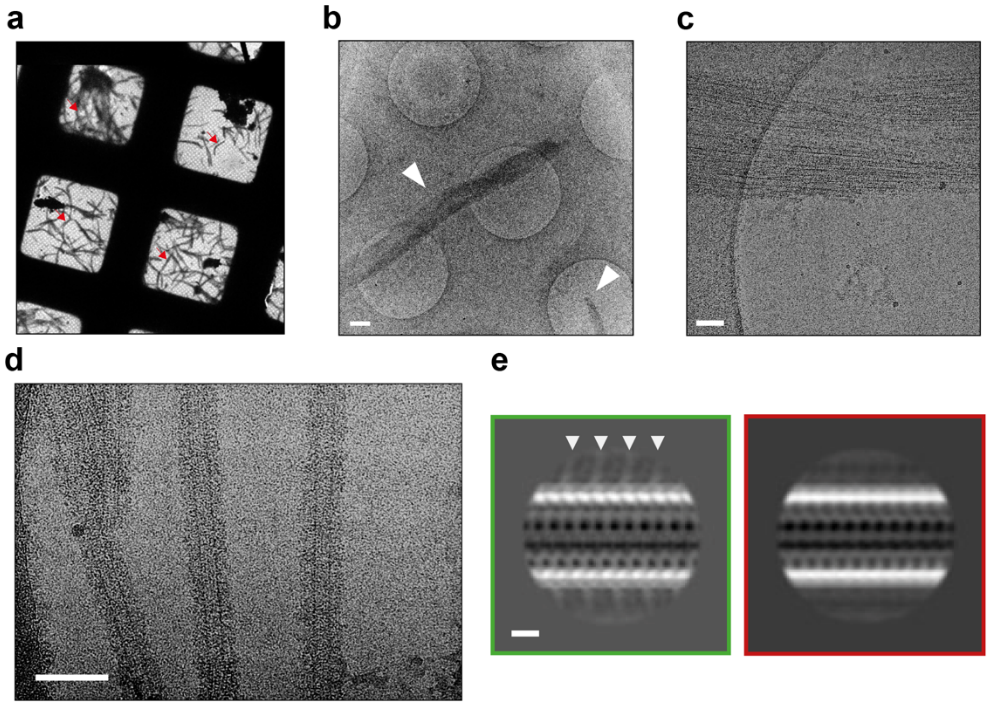

Supplementary Figure 7. Optimization of cryo-EM sample preparation for MTs decorated with *D. melanogaster* augmin N-clamp. a–c Representative sections of micrographs for *D. melanogaster* augmin N-clamp pre-incubated with MTs, showing the formation of large MT bundles at different scales. Scale bars: (b) 500 nm; (c) 100 nm. Arrowheads in a (red), and b (white) indicate MT bundles (n=1 experiment). **d** Representative section of cryo-EM micrograph showing MTs decorated with *D. melanogaster* augmin N-clamp after adsorption to the cryo-EM grid. Scale bar: 100 nm (n=1 experiment). **e** 2D class averages of a 14-PF MT decorated with *D. melanogaster* augmin N-clamp, displaying a class with regular decorating density corresponding to augmin N-clamp at every α/β-tubulin dimer (green, indicated by white arrowheads) and a class with no apparent decoration (red). Scale bar: 10 nm.

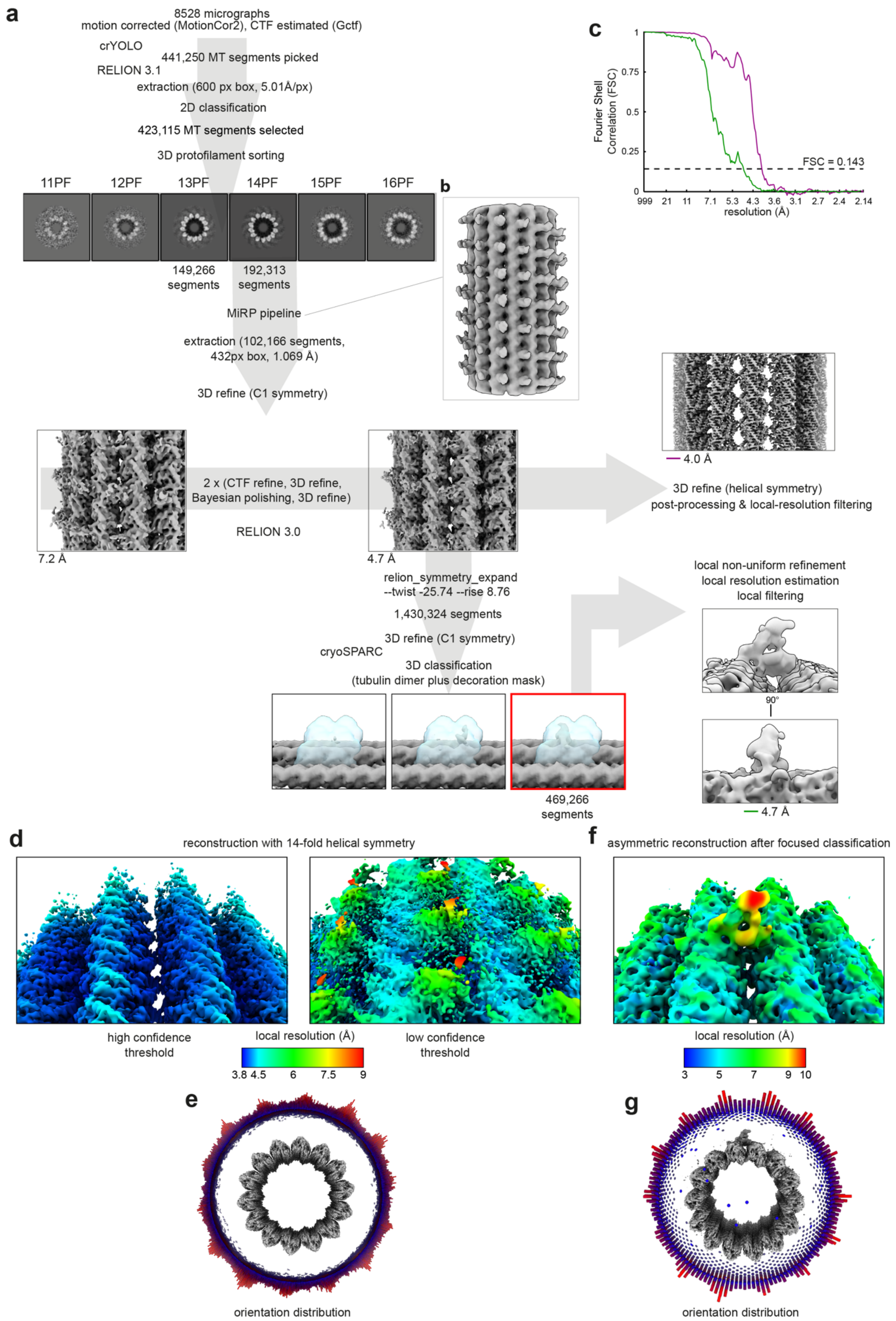

Supplementary Figure 8. Cryo-EM data processing of MTs decorated with wild-type *D. melanogaster* augmin N-clamp. a Cryo-EM processing workflow for MTs decorated with *D. melanogaster* augmin N-clamp. Selected 3D class is indicated with a red box. **b** 3D class obtained after the MiRP pipeline (v. 2) [3],

highlighting regular additional densities at every α/β-tubulin dimer. **c** Fourier Shell Correlation (FSC) curves for the 14-fold symmetric helical MT reconstruction (purple) and the C1 MT reconstruction after focused classification and refinement (green). **d** 14-fold symmetric helical MT reconstruction shown at high (*left*) and low (*right*) density threshold level colored according to local resolution. Coloring as indicated. **e** Orientation distribution of particles for the 14-fold symmetric helical MT reconstruction. **f** C1 reconstruction after focused classification and refinement colored according to local resolution. Coloring as indicated. **g** Orientation distribution of particles for the C1 reconstruction after focused classification and refinement, generated using the pyem package [4].

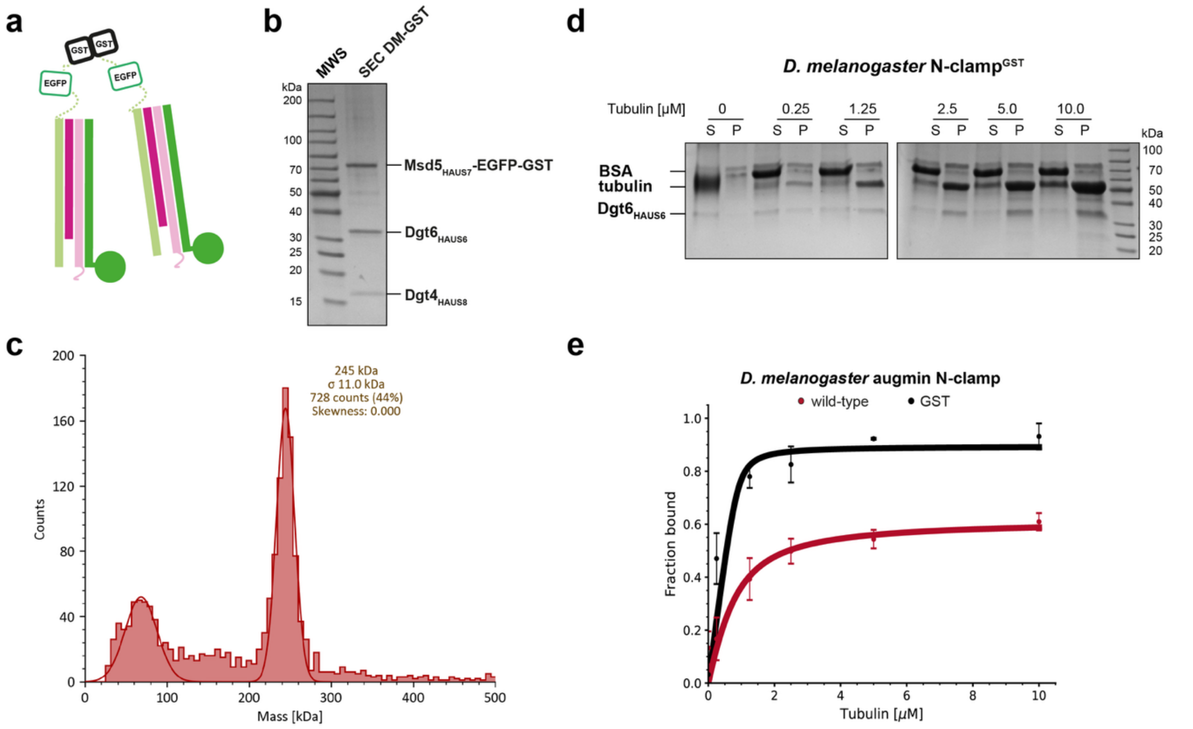

Supplementary Figure 9. GST-N-clamp dimers bind MTs with higher affinity. a Schematic representation of the *D. melanogaster* augmin GST-N-clamp dimer. Coloring as is Supplementary Figure 1. **b** Section of representative Coomassie-stained SDS-PAGE gel for purified augmin *D. melanogaster* augmin GST-N-clamp after SEC (DM-GST). * Msd1 was not visible due to its low molecular weight. MWS: Molecular weight standards. Purifications were repeated in n=3 experiments. **c** Histograms of mass photometry measurements of *D. melanogaster* augmin GST-N-clamp. Coloring as indicated (n=1 experiment). **d** Sections of representative SDS-PAGE gels for tubulin co-sedimentation assays of *D. melanogaster* GST-N-clamp compared to wild-type. Tubulin concentration is indicated at the top. Supernatant (S) and Pellet (P) fractions are indicated (see Table 1). **e** Pelleted fraction from tubulin co-sedimentation assays for *D. melanogaster* wild-type and augmin GST-N-clamp plotted against tubulin concentration. Shown as mean ± SD with fitted curves, n=3 repetitions. Source data are provided as a Source Data file.

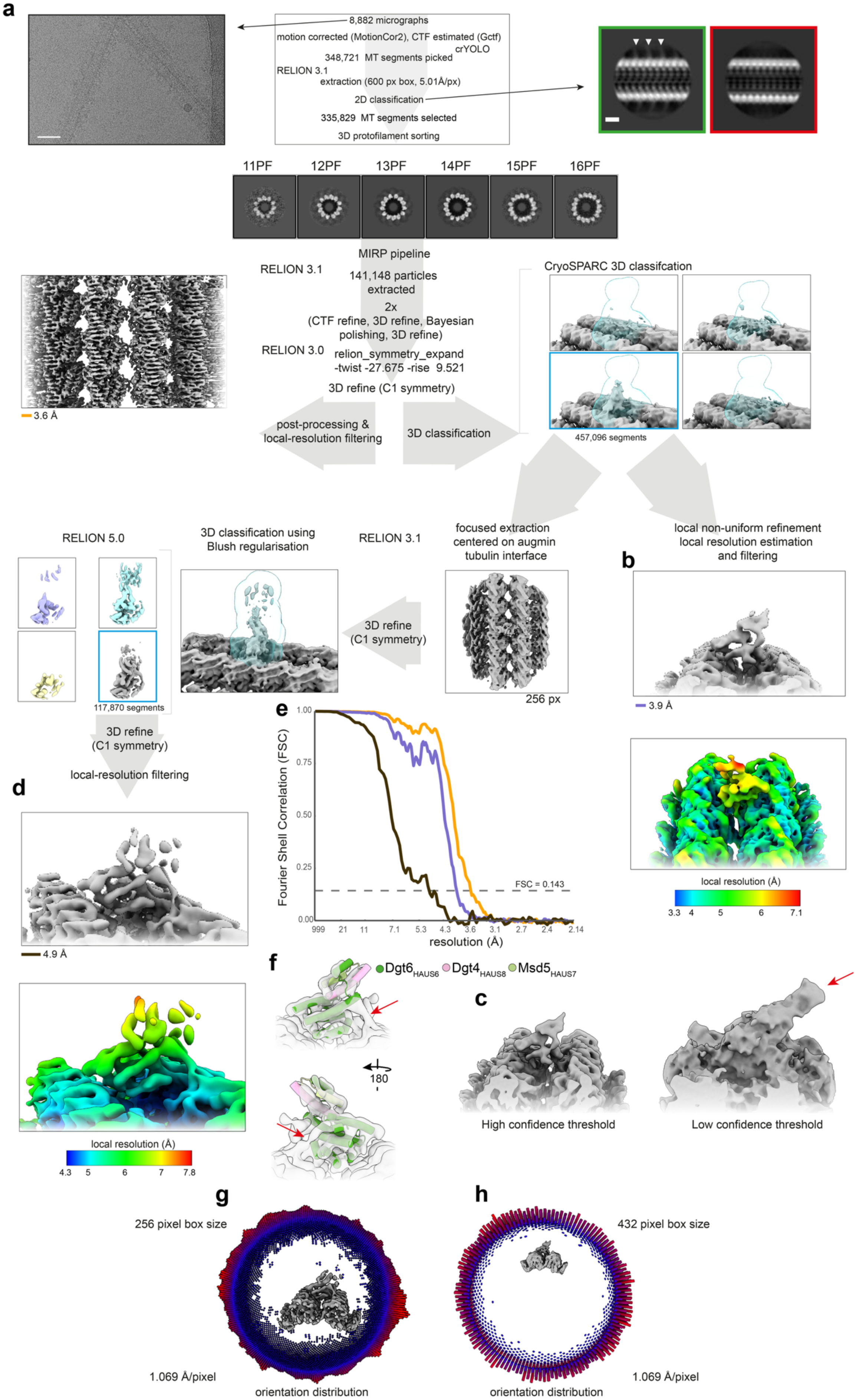
a
8,882 micrographs
motion corrected (MotionCor2), CTF estimated (Gctf)
crYOLO
348,721 MT segments picked
RELION 3.1
extraction (600 px box, 5.01Å/px)
2D classification
335,829 MT segments selected
3D protofilament sorting
11PF
12PF
13PF
14PF
15PF
16PF
MIRP pipeline
RELION 3.1
141,148 particles extracted
2x
(CTF refine, 3D refine, Bayesian polishing, 3D refine)
RELION 3.0
relion_symmetry_expand
-twist -27.675 -rise 9.521
3D refine (C1 symmetry)
3.6 Å
post-processing & local-resolution filtering
3D classification
CryoSPARC 3D classifcation
457,096 segments
focused extraction centered on augmin tubulin interface
local non-uniform refinement local resolution estimation and filtering
RELION 5.0
3D classification using Blush regularisation
RELION 3.1
3D refine (C1 symmetry)
256 px
117,870 segments
3D refine (C1 symmetry)
local-resolution filtering
b
3.9 Å
local resolution (Å)
3.3 4 5 6 7.1
e
Fourier Shell Correlation (FSC)
1.00
0.75
0.50
0.25
0.00
FSC = 0.143
999 21 11 7.1 5.3 4.3 3.6 3.1 2.7 2.4 2.14
resolution (Å)
d
4.9 Å
local resolution (Å)
4.3 5 6 7 7.8
f
Dgt6 HAUS6
Dgt4 HAUS8
Msd5 HAUS7
180
c
High confidence threshold
Low confidence threshold
g
256 pixel box size
1.069 Å/pixel
orientation distribution
h
432 pixel box size
1.069 Å/pixel
orientation distribution

Supplementary Figure 10. Cryo-EM data processing of MTs decorated with *D. melanogaster* augmin GST-N-clamp. a Cryo-EM processing workflow for MTs decorated with *D. melanogaster* augmin GST-N-clamp similar to Supplementary Figure 8. Left: Representative section of cryo-EM micrograph showing MTs decorated with augmin GST-N-clamp (n=1 experiment). Scale bar: 25 nm. Right**:** 2D class averages displaying a class with regular decorating density corresponding to augmin N-clamp at every α/β-tubulin dimer (green, indicated by white arrowheads) and a class with no apparent decoration (red). Scale bar: 10 nm. Middle: 3D classes which were further used are indicated with blue boxes. **b** Top**:** Particles from 3D classification were submitted to local resolution estimation and filtering and used to visualize α/β-tubulin distinction in Fig. 4c. Bottom: Density colored according to local resolution. **c** Comparison of the density from (b) at high (*left*) and low (*right*) density threshold level highlighting the more distal region of the augmin GST-N-clamp with an arrowhead (red). **d** Selected particles were further processed as depicted until final local resolution filtering of the final reconstruction shown in gray (*top*) and coloring according to local resolution (*bottom*). **e** Fourier Shell Correlation (FSC) curves for the reconstruction after symmetry expansion and C1 refinement (a, orange), after 3D classification in CryoSPARC (b, purple), and additionally processed in RELION 5 (d, black). **f** AF2 prediction of augmin N-clamp docked into the cryo-EM density shown in Fig. 4d. Red arrowheads indicate unassigned density segments that extend from the tubulin E-hook regions. Coloring as indicated. **g** Orientation distribution of particles for reconstruction shown in d. **h** Orientation distribution of particles for reconstruction shown in b, generated using the pyem package [4].

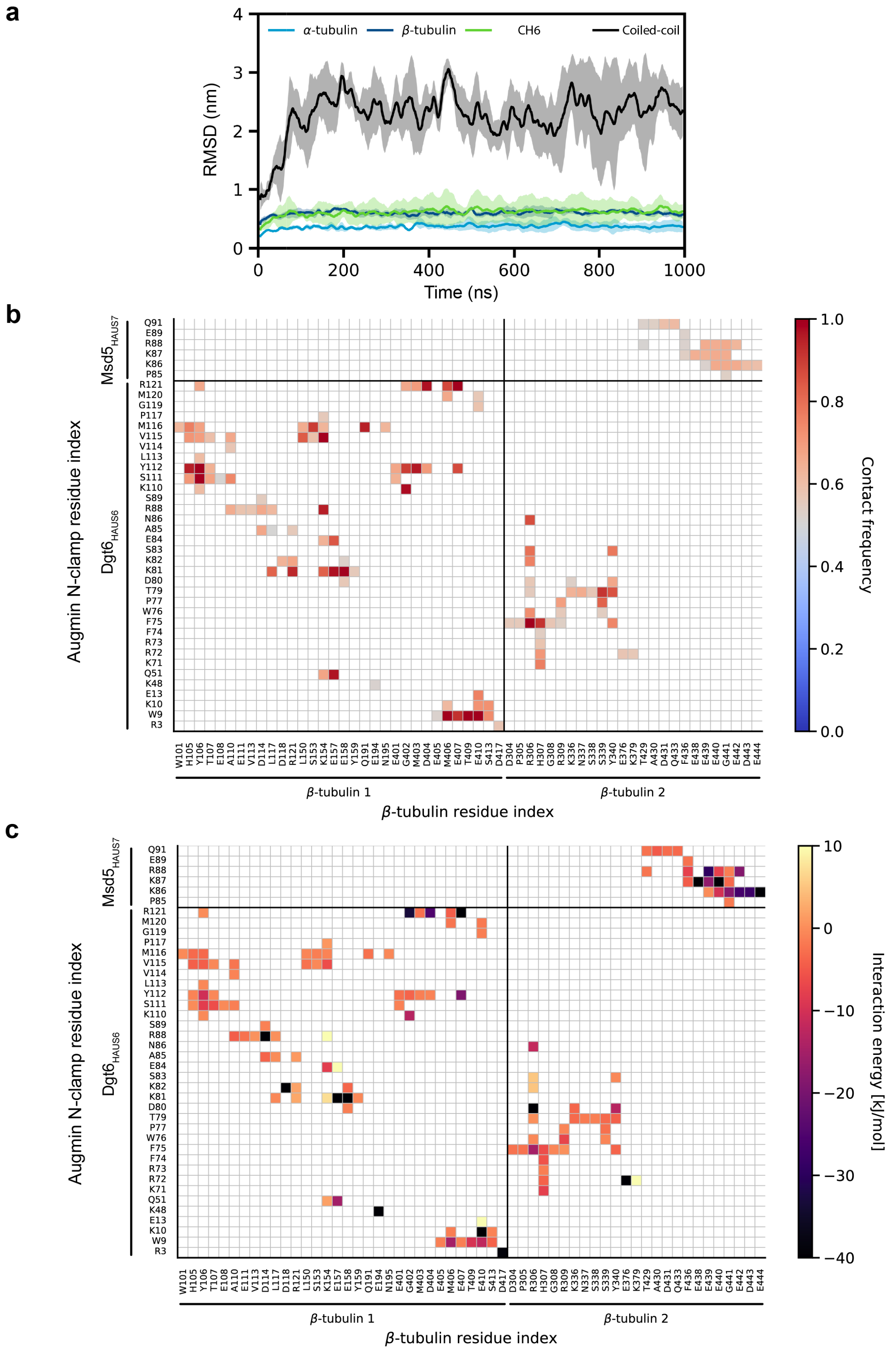

Supplementary Figure 11. Molecular characterization of the β-tubulin/augmin N-clamp interface via atomistic Molecular Dynamics simulations over the last 500 ns. a Root-mean-square deviation (RMSD) of unrestrained α-tubulins and β-tubulins interacting with augmin N-clamp, the hammerhead, and the distal coiled-coil over the full simulation time (1000 ns), averaged across three independent trajectories (n = 3). Data are presented

as mean ± SD. Coloring as indicated. **b** Pairwise contact map highlighting *D. melanogaster* augmin N-clamp residues that interact with the two β-tubulin monomers for ≥50% of the total simulated cumulative time. A contact is defined when any atom pair between the *D. melanogaster* augmin N-clamp and β-tubulin is within 0.6 nm. The analysis is based on the final 500 ns of each trajectory (n=3) and represents averaged data. **c** Pairwise interaction energy map highlighting N-clamp residues interacting with the two β-tubulin monomers, displaying the average interaction energy as the sum of electrostatic and van der Waals contributions, based on the contacts identified in (b).

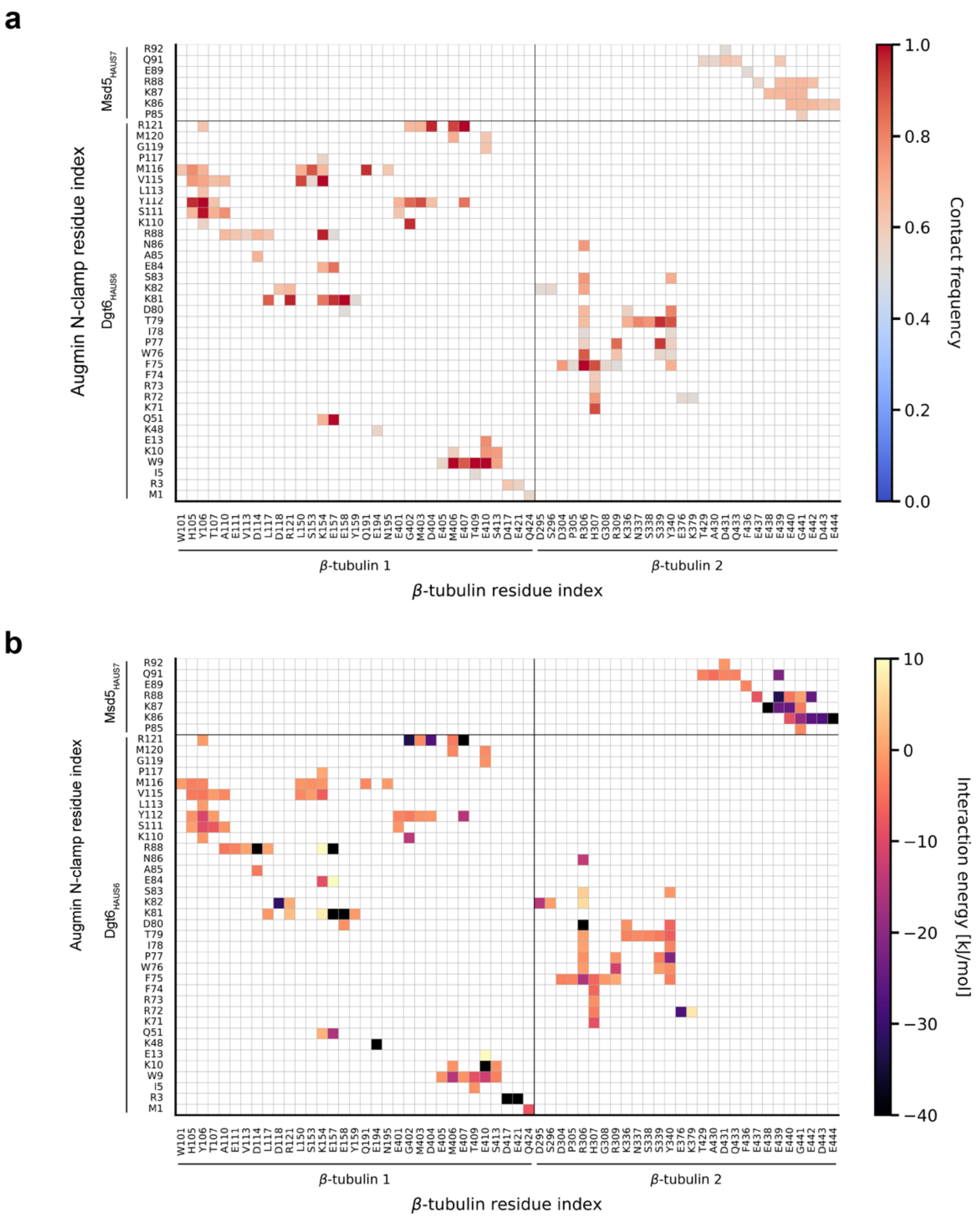

Supplementary Figure 12. Molecular characterization of the β-tubulin/augmin N-clamp interface via atomistic molecular dynamics simulations over the last 200 ns. a Pairwise contact map highlighting *D. melanogaster* augmin N-clamp residues that interact with the two β-tubulin monomers for ≥50% of the total simulated cumulative time. A contact is defined when any atom pair between the *D. melanogaster* augmin N-clamp and β-tubulin is within 0.6 nm. The analysis is based on the final 200 ns of each trajectory (n=3) and represents averaged data. **b** Pairwise interaction energy map highlighting N-clamp residues interacting with the two β-tubulin monomers, displaying the average interaction energy as the sum of electrostatic and van der Waals contributions, based on the contacts identified in (a).

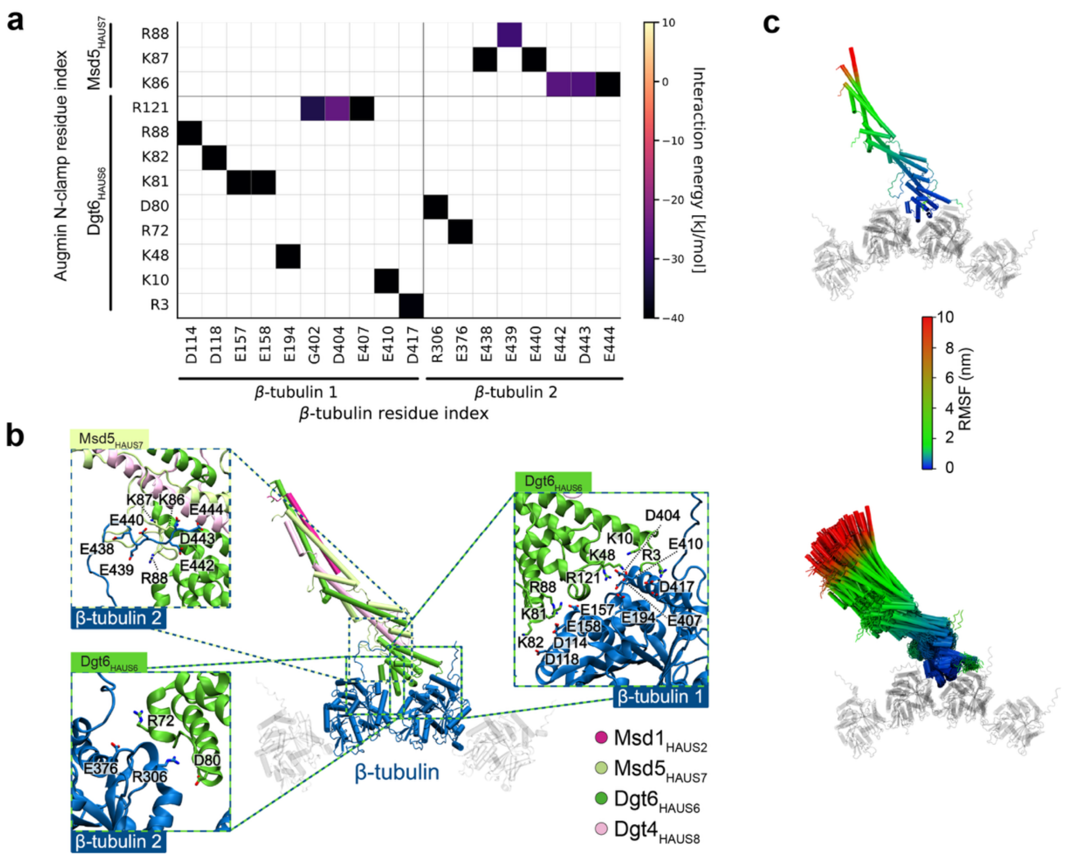

Supplementary Figure 13. Visualization of the β-tubulin/augmin N-clamp interface analyzed via atomistic Molecular Dynamics simulations over the last 500 ns. a Pairwise contact map highlighting *D. melanogaster* augmin N-clamp residues that interact with the two β-tubulin monomers for ≥50% of the total simulated cumulative time. A contact is defined when any atom pair between the *D. melanogaster* augmin N-clamp and β-tubulin is within 0.6 nm. The analysis is based on the final 500 ns of each trajectory (n=3) and represents averaged data. **b** Representative structure of the MT-bound *D. melanogaster* augmin N-clamp, with close-up views highlighting all residues identified as involved in contacts. Key interacting residues with an interaction energy of less than -20 kJ/mol, as indicated by a dark purple color in panel (a), are labeled. Coloring as indicated. **c** Top: Root-Mean-Square-Fluctuation (RMSF) of the MT-bound N-clamp, with the color gradient representing the degree of fluctuations (in nm) along the structure. Blue regions indicate minimal fluctuation, while red regions emphasize higher flexibility. *Bottom*: Overlay of 100 frames visualizing the actual movement. The RMSF is calculated for the entire trajectory of each run and subsequently averaged across all runs, coloring is indicated.

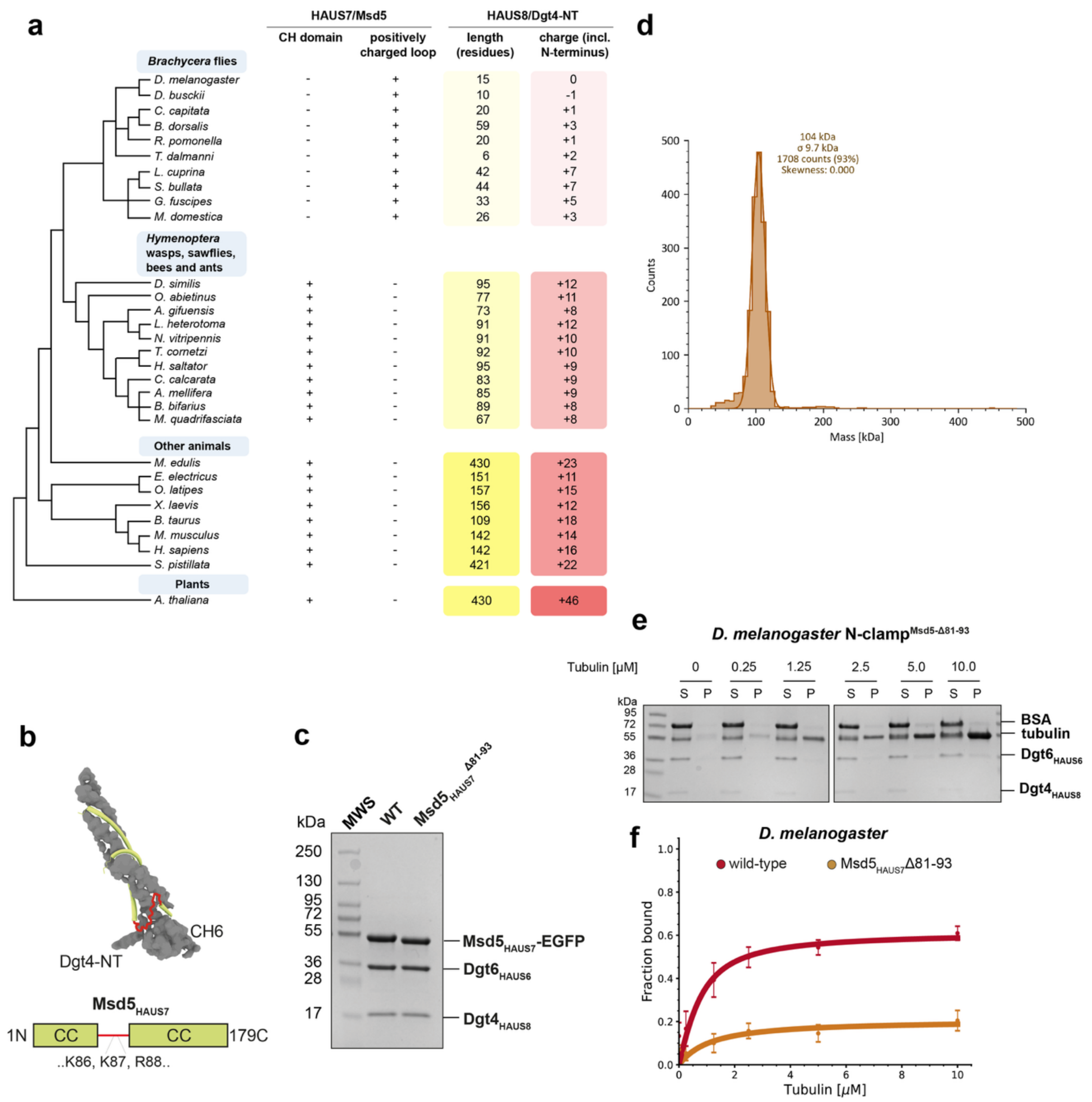

Supplementary Figure 14. Msd5$_{HAUS7}$ loop deletion impacts MT binding. a Comparison of the presence of a CH7 domain and/or a positively charged loop analogous to the one identified in *D. melanogaster* for HAUS7/Msd5 as well as the length (yellow) and net charge (red) of the HAUS8/Dgt4-NT for different species. Schematic representation illustrates evolutionary relationship between species. **b** Schematic representation of the *D. melanogaster* augmin N-clamp highlighting Msd5$_{HAUS7}$ (green) and the location of the deleted loop region (red). **c** Section of representative Coomassie-stained SDS-PAGE gel for purified *D. melanogaster* wild-type (WT) and Msd5$_{HAUS7}$Δ81-93 augmin N-clamps. * Msd1 was not visible due to its low molecular weight. MWS: Molecular weight standards. Purifications were repeated in n=3 experiments. **d** Histogram of mass photometry measurements for *D. melanogaster* Msd5$_{HAUS7}$Δ81-93 augmin N-clamp (n=1 experiment). Coloring as indicated. **e** Sections of representative SDS-PAGE gels for tubulin co-sedimentation assays of *D. melanogaster* Msd5$_{HAUS7}$Δ81-93 augmin N-clamps. Tubulin concentration is indicated on the top. Supernatant (S) and Pellet (P) fractions are indicated (see Table 1). **f** Pelleted fraction from tubulin co-sedimentation assays for *D. melanogaster* wild-type and Msd5$_{HAUS7}$Δ81-93 augmin N-clamp plotted against tubulin concentration. Shown as mean ± SD (see Methods and Table 1) with fitted curves. Curves of the wild-type complexes from Fig.1. Experiments with Msd5$_{HAUS7}$Δ81-93 constructs were performed in n=3 repetitions. Source data are provided as a Source Data file.

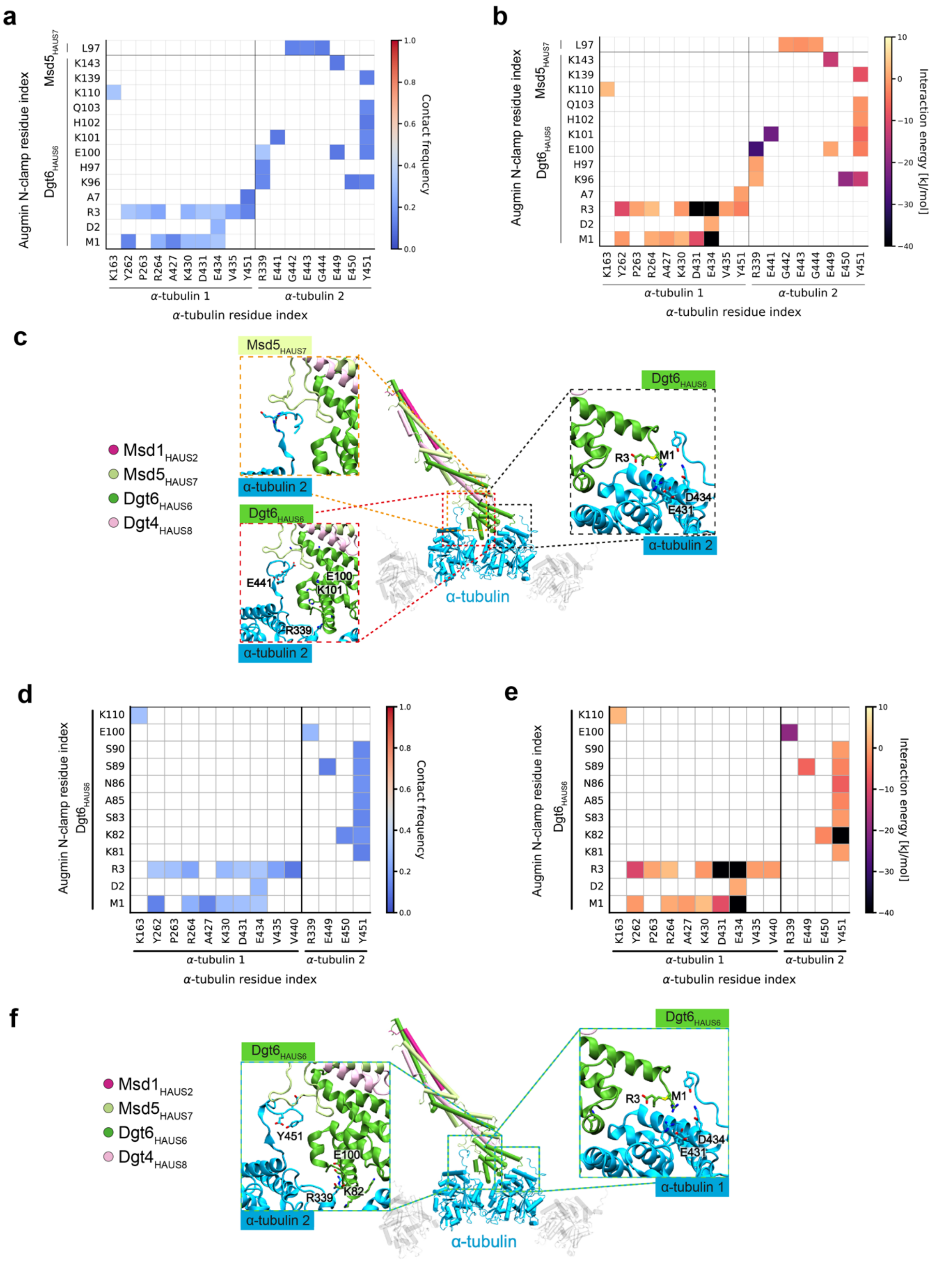

Supplementary Figure 15. Molecular characterization of the α-tubulin/augmin N-clamp interface via atomistic Molecular Dynamics simulations over the last 200 and 500 ns. a Pairwise contact map highlighting *D. melanogaster* augmin N-clamp residues that interact with the two α-tubulin monomers for ≥50% of the total simulated cumulative time. A contact is defined when any atom pair between the augmin N-clamp and α-tubulin is within 0.6 nm. The analysis is based on the final 200 ns of each trajectory (n=3) and represents averaged data. **b**

Pairwise interaction energy map highlighting *D. melanogaster* augmin N-clamp residues interacting with the two α-tubulin monomers, displaying the average interaction energy as the sum of electrostatic and van der Waals contributions, based on the contacts identified in panel (a). **c** Representative structure of the MT-bound *D. melanogaster* augmin N-clamp complex, with close-up views highlighting all residues identified as involved in contacts. Key interacting residues with an interaction energy of less than -20 kJ/mol, as indicated by a dark purple color in panel (b), are labeled. Coloring as indicated. **d** Pairwise contact map highlighting *D. melanogaster* augmin N-clamp residues that interact with the two α-tubulin monomers for ≥50% of the total simulated cumulative time. A contact is defined when any atom pair between the augmin N-clamp and α-tubulin is within 0.6 nm. The analysis is based on the final 500 ns of each trajectory (n=3) and represents averaged data. **e** Pairwise interaction energy map highlighting *D. melanogaster* augmin N-clamp residues interacting with the two α-tubulin monomers, displaying the average interaction energy as the sum of electrostatic and van der Waals contributions, based on the contacts identified in panel (d). **f** Representative structure of the MT-bound *D. melanogaster* augmin N-clamp complex, with close-up views highlighting all residues identified as involved in contacts. Key interacting residues with an interaction energy of less than -20 kJ/mol, as indicated by a dark purple color in panel (e), are labeled.

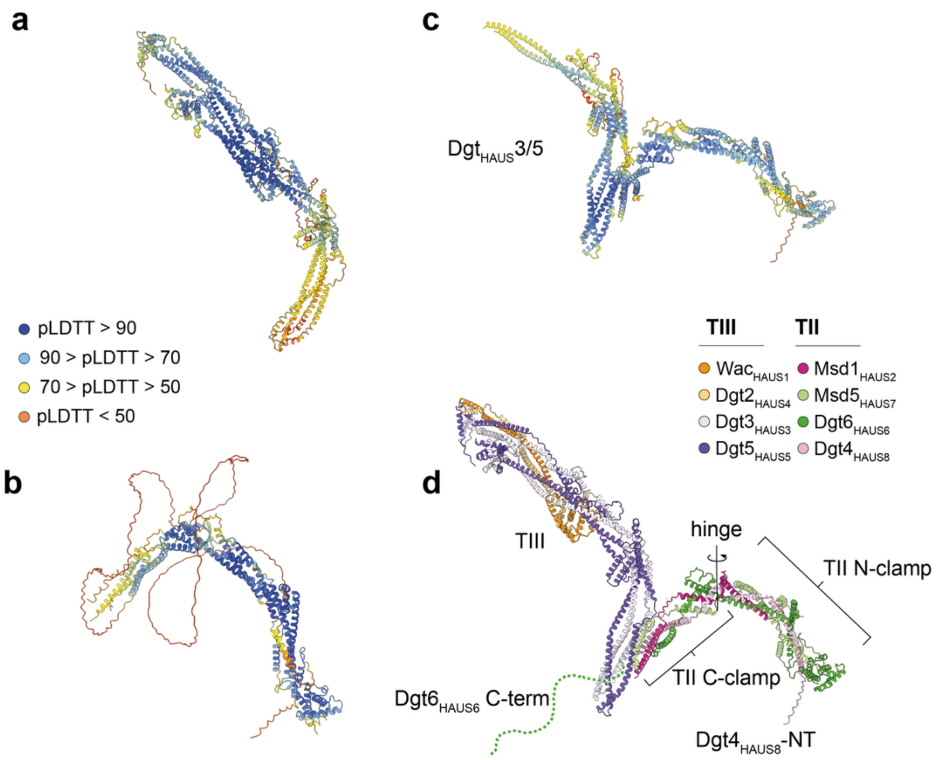

Supplementary Figure 16. Composite AF2 model of *D. melanogaster* augmin holocomplex. a-c Highest-scoring models of *D. melanogaster* augmin TIII tetramer (**a**), TII tetramer (**b**) and TII + Dgt$_{HAUS}$3/5 (**c**) predicted by AF2 and colored by pLDDT as indicated. **d** Molecular model of the *D. melanogaster* augmin holocomplex obtained by merging the highest-scoring models of TIII and TII + Dgt$_{HAUS}$3/5 predicted by AF2 shown in panels (**a**,**c**). Coloring as indicated. Predicted Aligned Error (PAE) plots are shown in Supplementary Figure 19. Sequences used for AF2 predictions are listed in Supplementary Table 7.

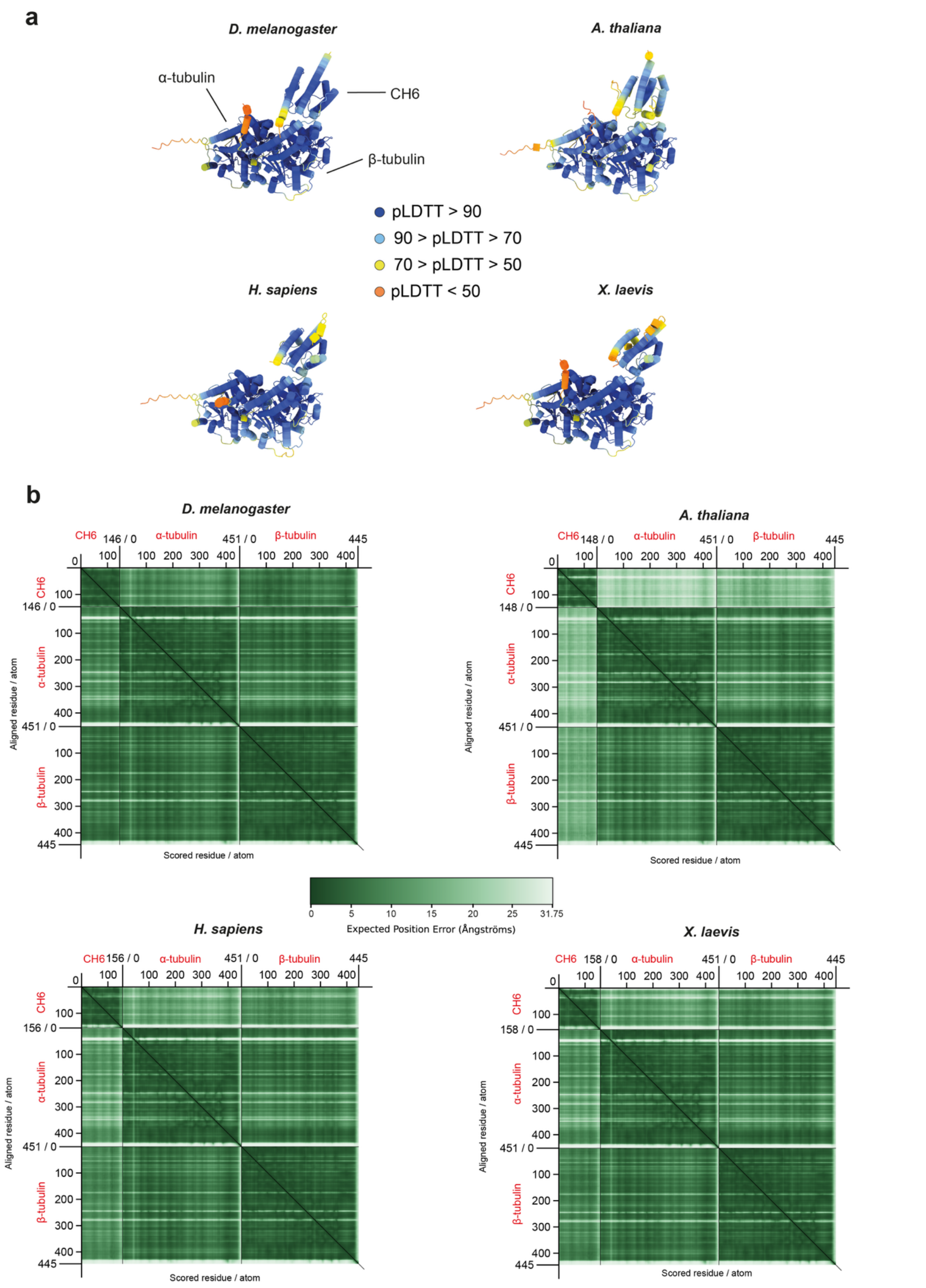

Supplementary Figure 17. AF2 predictions for α/β-tubulin in complex CH6 domains for different organisms. a First-ranked AF2 prediction of CH6 domains from different organisms with porcine α/β-tubulin, colored according to pLDDT score. Coloring is indicated. **b** Predicted Aligned Error (PAE) plots of the first-ranked

model for AF2 predictions shown in panel (a). Coloring is as indicated. Plots were generated using the PAE viewer tool[5]. Sequences used for AF2 predictions are listed in Supplementary Table 7.

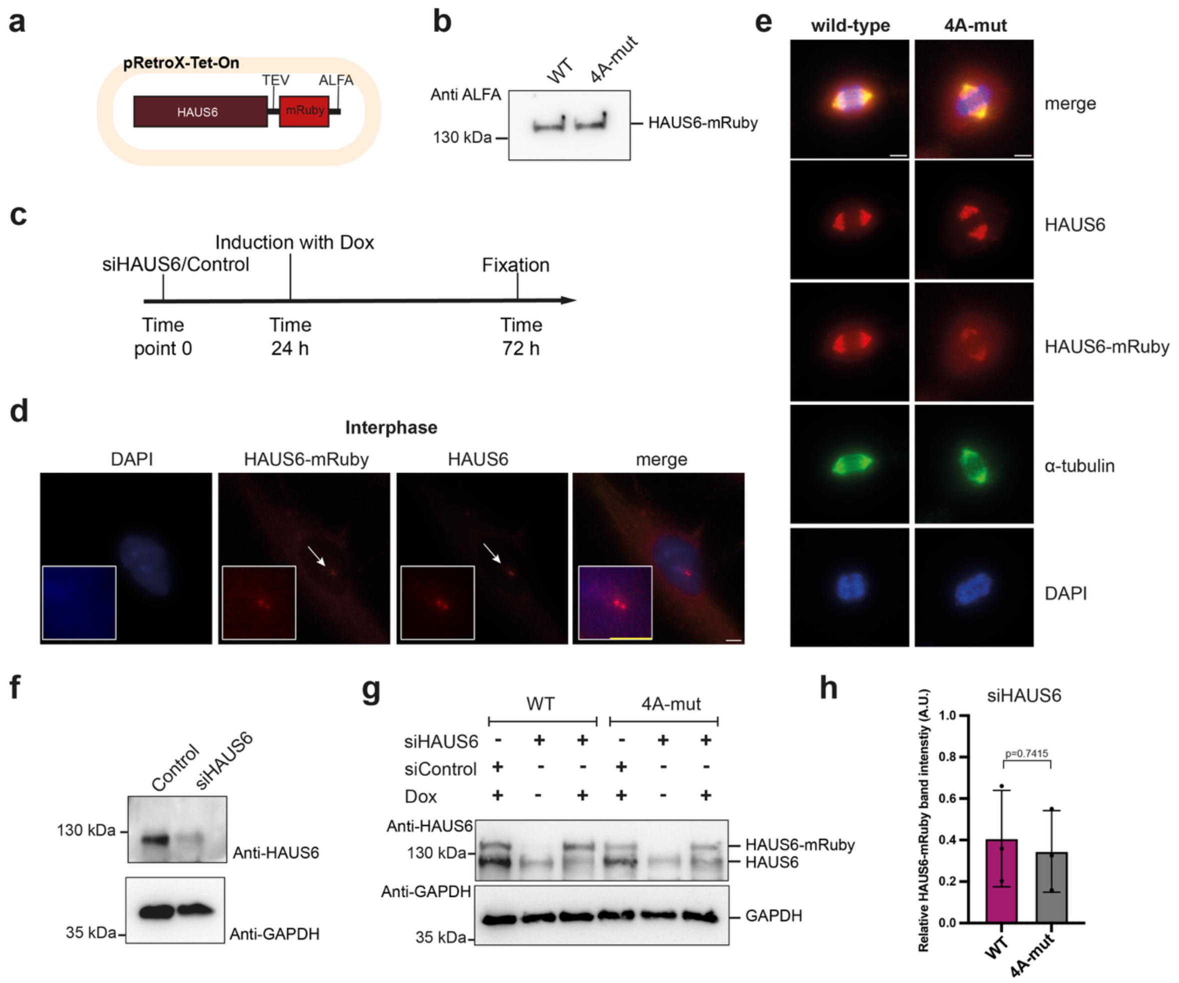

Supplementary Figure 18. Function of the CH6 domain in RPE1 cells. a Diagram of pRetroX-Tet-On *HAUS6* constructs, stably integrated into the genome and designed for expression in RPE1 cells. **b** Representative section of immunoblot analysis of RPE1 cells showing expression of *HAUS6*-mRuby wild-type (WT) or *HAUS6*$^{4A\text{-}mut}$ (4A)-mRuby after induction with 20 ng/ml of doxycycline (Dox). Detection via Anti-ALFA antibody (n=3 experiments). **c** Workflow for siRNA experiments in RPE1 cells. **d** Representative immunofluorescence image of *HAUS6*$^{4A\text{-}mut}$-mRuby mutant cells in interphase showing clear HAUS6-mRuby and HAUS6 localization at the centrosome (n=3 experiments). Cells were treated with non-specific control siRNA (siControl) and induced with 20 ng/ml Dox. Cells were stained for α-tubulin, DAPI, and HAUS6, with additional imaging of the HAUS6-mRuby channel. White boxes are zooms of the centrosome. Scale bars (white and yellow): 5 µm. **e** Representative immunofluorescence images of wild-type and 4A-mut cells in anaphase, showing HAUS6-mRuby localization on spindle MTs (n=3 experiments). Cells were treated with non-specific control siRNA (siControl) and expression of *HAUS6*-mRuby wild type or 4A-mut was induced with 20 ng/ml Dox. Cells were stained for α-tubulin, DAPI, and HAUS6, with additional imaging of the HAUS6-mRuby channel. Scale bars: 5 µm. **f** HAUS6 siRNA (siHAUS6) knockdown was examined via immunoblot analysis of RPE1 wild-type cells, using anti-HAUS6 and anti-GAPDH antibodies. **g** Representative section of immunoblot analysis for the siHAUS6 experiment (Fig. 6d,e) stained with anti-HAUS6 and anti-GAPDH antibodies. **h** Quantification of HAUS6-mRuby expression levels in the siHAUS6 knockdown condition. Band intensities of immunoblot analysis for the HAUS6-mRuby bands relative to the GAPDH bands were quantified for wild-type *HAUS6*^{WT} (purple) and *HAUS6*$^{4A\text{-}mut}$ (gray) cells upon siHAUS6 knockdown and induction with 20 ng/ml Dox. The bars represent mean ± SD. Experiments were repeated and quantified from three independent experiments (n = 3). *P-value* is indicated and determined via two tailed t-test. Source data are provided as a Source Data file.

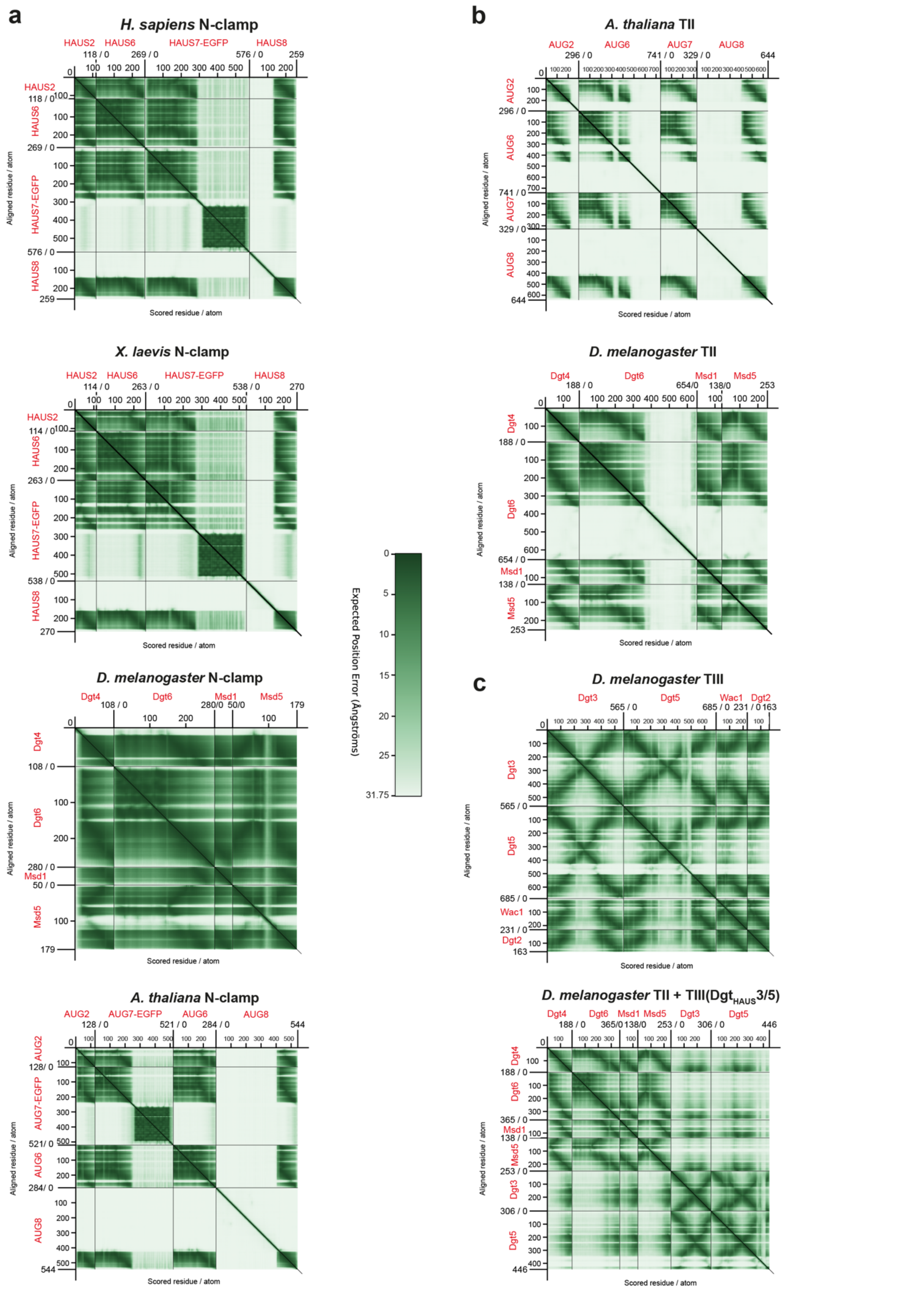

Supplementary Figure 19. Quality metrics for AF2 predictions of augmin subcomplexes. a-c Predicted Aligned Error (PAE) plots of first-ranked AF2 predictions for augmin N-clamps in Fig.1c (**a**), augmin TII in Supplementary Figure 1 (**b**) and subcomplexes used for preparing the *D. melanogaster* augmin holocomplex model in Supplementary Figure 16 (**c**). Coloring as indicated. Plots were generated using the PAE viewer tool [5].

Supplementary Tables

Supplementary Table 1: MS list of *X. laevis* augmin N-clamp

Protein name	Unique/total peptides
HAUS7-EGFP	53/144
HAUS8	38/122
HAUS6	49/119
HAUS2	12/51

Supplementary Table 2: MS list of *H. sapiens* augmin N-clamp

Protein name	Unique/total peptides
HAUS7-EGFP	42/70
HAUS8	50/50
HAUS6$^{4A\text{-}mut}$	10/51
HAUS2	16/16

Supplementary Table 3: MS list of *A. thaliana* augmin N-clamp

Protein name	Unique/total peptides
AUG7$_{HAUS7}$*-EGFP*	64/147
AUG8$_{HAUS8}$	23/41
AUG6$_{HAUS6}$	27/69
AUG2$_{HAUS2}$	16/42

Supplementary Table 4: MS list of *D. melanogaster* augmin N-clamp

Protein name	Unique/total peptides
Msd5$_{HAUS7}$*-EGFP*	45/75
Dgt6$_{HAUS6}$	53/53
Dgt4$_{HAUS8}$	11/11
Msd1$_{HAUS2}$	4/4

Supplementary Table 5: Details for augmin subunits used for experiments including identifier (NCBI (NP_) or Uniprot-ID), amino acid ranges used for expression and segments that were removed in deletion constructs.

Species	Gene Fragment	Identifier	N-clamp sequence length (AA)	HAUS8-ΔN (deleted AA)
H. sapiens	HAUS2	NP_060567.1	1-118	
	HAUS6	NP_060115.3	1-269	
	HAUS7	NP_001372411.1	1-299	
	HAUS8	NP_219485.1	1-248	1-138
X. laevis	HAUS2	NP_001085195.1	1-114	
	HAUS6	NM_001097095.1	1-263	
	HAUS7	NP_001121229.1	1-261	
	HAUS8	NP_001090302.1	1-259	1-155
A. thaliana	AUG2	NP_565760.1	1-128	
	AUG6	NP_568585.1	1-284	
	AUG7	NP_568354.1	1-241	
	AUG8	NP_001329921.1	1-533	1-409
D. melanogaster	Msd1	Q9W0G7	1-50	
	Dgt6	Q9VAP2	1-280	
	Msd5	Q9W0G6	1-179	
	Dgt4	Q9W4M8	1-108	1-20

Supplementary Table 6: List of oligonucleotides.

Primer Name	Sequence (5' to 3')	Source	Identifier
Duet_side1_PL_fwd	TGAGAATTCGAGCTCGGC	Würtz et al	N/A
Duet_side1_PL_rev	CATGGTATATCTCCTTCTTAAAGTTAAC	Würtz et al	N/A
Duet_side2_PL_fwd	CTGTCCGGCCATCATCAC	Würtz et al	N/A
Duet_side2_PL_rev	CATATGTATATCTCCTTCTTATACTTAACTAATATACTAAG	Würtz et al	N/A
XL_HAUS2_fwd	taagaaggagatataccatgATGGCAGCTAACCCGTGG	Gao et al[6]	N/A
XL_HAUS2_fwd	gcgccgagctcgaattctcaTTGAGCCAAGCGTTGCCTC	Gao et al[6]	N/A
XL_HAUS6_fwd	taagaaggagatataccatgCAATCGGGATCCAGGCCAC	Gao et al[6]	N/A
XL_HAUS7_fwd	aagaaggagatatacatatgACCGGAGGAAAAGAGTTG	Gao et al[6]	N/A
XL_HAUS7_rev	tggtgatgatggccggacagTTGGAATTCATTTGCGTAG	Gao et al[6]	N/A
XL_HAUS6_rev	gcgccgagctcgaattctcaACGCACAACGGCGTCTAC	Gao et al[6]	N/A
XL_HAUS8_fwd	aagaaggagatatacatatgTCGGAAGCTGGAGTTGCTC	Gao et al[6]	N/A

XL_HAUS8_rev	tggtgatgatggccggacagATGTCTAGTAGAGTCCAGGGC	Gao et al[6]	N/A
HS_HAUS8_fwd	aagaaggagatatacatatgGCGGATTCCTCGGGGCGAG	Gao et al[6]	N/A
HS_HAUS8_rev	tggtgatgatggccggacagGGGCAGCTCGTGCCTGGT	Gao et al[6]	N/A
HS_HAUS6_fwd	taagaaggagatataccatgAGCTCGGCCTCGGTCACC	Gao et al[6]	N/A
HS_HAUS6_rev	gcgccgagctcgaattctcaTCCATCTAAAGCATATTGGTTAACAAGACTAAGGACC	Gao et al[6]	N/A
HS_HAUS7_fwd	aagaaggagatatacatatgGCGGGGCAGGACGCTGGC	Gao et al[6]	N/A
HS_HAUS7_rev	tggtgatgatggccggacagCTGGATGATGGGGCCGCACG	Gao et al[6]	N/A
HS_HAUS2_fwd	taagaaggagatataccatgGCCGCTGCCAACCCGTGG	Gao et al[6]	N/A
HS_HAUS2_rev	gcgccgagctcgaattctcaGCACATGGGTTTCAACAGTCTTTGCCTAAG	Gao et al[6]	N/A
Pet26-EGFP_conversion_PL_H_fwd	ccatcatccagctgtccggcGAAAACCTGTATTTTCAGGGC	This study	N/A
Pet26-EGFP_conversion_PL_general_rev	TCACCGATGGGGAAGATC	This study	N/A
Pet26-EGFP_conversion_insert_general_fwd	ccgatcttccccatcggtgaCCGGCGTAGAGGATCGAG	This study	N/A
Pet26-EGFP_conversion_insert_H_rev	GCCGGACAGCTGGATGATG	This study	N/A
Pet26-EGFP_conversion_PL_XL_fwd	atgaattccaactgtccggcGAAAACCTGTATTTTCAGGGC	This study	N/A
Pet26-EGFP_conversion_insert_XL_rev	GCCGGACAGTTGGAATTC	This study	N/A
HHAUS8_Ndel_fwd	aagaaggagatatacatatgAGCCCGGATTTATCTGAAG	This study	N/A
AT_AUG8_Ndel_fwd	aagaaggagatatacatatgACAAGTGTGTTGTCCTTTATC	This study	N/A
HAUS8_General-Ndel_rev	CATATGTATATCTCCTTCTTATACTTAAC	This study	N/A
Dgt4 -N-del _fwd	tataagaaggagatatacatATGGACGACATTCAGTATC	This study	N/A
Duet_combination_PL_fwd	TGAGCAATAACTAGCATAACC	This study	N/A
Duet_combination_PL_rev	ATGTGCTGGCGTTCAAATTTC	This study	N/A

Pet26_combination_insert_fwd	aaatttgaacgccagcacatTAGAAATAATTTTGTTTAACTTTAAGAAGGAG	This study	N/A
Pet26_combination_insert_rev	gttatgctagttattgctcaCTTGTACAGCTCGTCCATG	This study	N/A
hK84909198A_f	CTGAATTCGCAGCCCATTGCTGTGAATGGATAGCAAGGATTTCTGGTGAATGTGGAAG	This study	N/A
hK84909198A_r	AGCAATGGGCTGCGAATTCAGTGTCACTTGCTTGCTCAAATGGGGGCCAA	This study	N/A
HAUS6_SI_resi_f	TCGGTGAAGCAAGTAAGGAACTTGAGATCTGAATGTATAG	This study	N/A
HAUS6_SI_resi_r	TCCTTACTTGCTTCACCGATAATTGTGCATTTTCCTGATATTTTTG	This study	N/A
HAUS6-FL-MB_formodiN-clamp_PL _f	GCTTTAGATGGAACTAATGTTG	This study	N/A
HAUS6-FL-MB_formodiN-clamp_PL _r	ATTTATAGGTTTTTTTATTACAAAACTG	This study	N/A
Modi-N-clamp_insert _f	tgtaataaaaaaacctataaatATGAGCTCGGCCTCGGTC	This study	N/A
Modi-N-clamp_insert _r	acattagttccatctaaagcATATTGGTTAACAAGACTAAGGACCGAAC	This study	N/A
pMT_GFP_fwd	GGCGGCCGCTCGAGGGAG	This study	N/A
pMT_GFP_rev	AAGGGCAATTCCACCACACTGGACTAG	This study	N/A
Dgt6_NT_fwd	agtgtggtggaattgcccttATGGATCGTACTATTATCGC	This study	N/A
Dgt6_NT_rev	cggcatcgcaATCAAATAACACGCGCATC	This study	N/A
DM_ct_fwd	gttatttgatTGCGATGCCGTGACCGAG	This study	N/A
DM_ct_rev	atctccctcgagcggccgccCTCTGTTAGTCGTCGACGCAAC	This study	N/A
Msd5-deltaloop_fwd	ttgaagaatatcccgcaaatACAAATAGTACTTTGTTGCG	This study	N/A
Msd5-deltaloop_rev	ATTTGCGGGATATTCTTC	This study	N/A
27-GFP+GST_PL_fwd	GGGCCCCACCACCATCAC	This study	N/A
27-GFP+GST_PL_rev	CAGAACTTCCAGCTTGTACAGCTC	This study	N/A
GST_fwd	tgtacaagctggaagttctgATGTCCCCTATACTAGGTTATTG	This study	N/A
GST_rev	tggtgatggtggtggggcccTTTTGGAGGATGGTCGCC	This study	N/A
dgt6_2_fw_qPCR	TGGCCTTCGTGCATGTGAT	This study	N/A
dgt6_2_rv_qPCR	GCTTCTCGTTCAGATGCTTCAG	This study	N/A

Gapdh_fw_qPCR	GCT CCG GGA AAA GGA AAA	This study	N/A
Gapdh_rv_qPCR	TCC GTT AAT TCC GAT CTT CG	This study	N/A
Dgt6_fw_dsRNA	TAATACGACTCACTATAGGGAGAGCCCGGAGGCATGAGG	This study	N/A
Dgt6_rv_dsRNA	TAATACGACTCACTATAGGGAGATCCTTCTGTGACTGCATCA	This study	N/A
Brown_fw_dsRNA	TAATACGACTCACTATAGGGAGCTCTCCTTCGTGCCCGT	This study	N/A
Brown_rv_dsRNA	TAATACGACTCACTATAGGGATCAATAGTAACCACTGCGGTGAAT	This study	N/A
Dgt6_dsRNA	TCCTTCTGTGACTGCATCACAGTCGAACAGCACTCGCATGCCCCGAAGTGCCTTCTCCACCGCCTCCTTGCTCTGGTTGTTCTCGGCTTGCTTCACCTGGAAAAGATCGATGGCCTCCTTGAGACCGCACAGAGATGCCTCCAGGTCGAATTTCCTTTCGGTGGGTTGGGTAATGACCCGCTCCACGCCGAGTGCAGCGGTGGCCTCGAACTCATCCAGAAAGCCATCGTCCGCCAGTTGCTCCTCGGGCACTCCCATGTCTGCAGACAGATCGGCCATCAGGCGTCTGATCTTCTGGGTCTTGTCCCGCAACAGGGCCGTATTCTCCTCCAGATTGACCACATACGAAGAAGCGTACTCCTTCATCACGGCATTTTGGCGGGCCATGACCTTGGCGCTCACGTTTGGTGTTCCCGCCTCTAAGCCAAGTGACTTCTCGCGCTGCTTGATGAGCTCCTGGATCACGAAGCCCACGAACTCGAGCAGGAAGTTGATGAACCTCATGCCTCCGGGC	This study	N/A
Brown_dsRNA	GCTCTCCTTCGTGCCCGTGGCCTTCTTCAAGGGCTACGTCTTCCTGTCGGTGATATATGCCTCCATATACTACACGCGCGGCTTCCTTTTGTACCTCAGCATGGGCTTCCTGATGAGCCTGTCCGCGGTGGCGGCTGTTGGCTATGGGGTCTTCCTCTCCAGCCTCTTCGAGTCGGATAAGATGGCCTCCGAGTGCGCGGCGCCCTTCGATCTGATCTTCCTTATCTTTGGCGGCACCTACATGAATGTGGACACAGTGCCTGGACTAAAGTACCTCTCGTTGTTCTTCTACTCCAACGAGGCGCTGATGTACAAGTTCTGGATCGACATCGATAATATCGACTGCCCCGTCAACGAGGATCATCCGTGCATCAAGACCGGAGTGGAGGTGCTGCAGCAGGGATCCTATCGCACCGCCGACTACACCTATTGGCTGGACTGCTTCAGTCTGGTGGTGGTGGCCGTCATCTTTCACATCGTGTCCTTTGGGCTGGTTAGGCGATACATTCACCGCAGTGGTTACTATTGA	This study	N/A

Supplementary Table 7: Details AF2 predictions performed during the study including species, identifier (NCBI (NP_) or Uniprot-ID), amino acid ranges and AF2 version used for prediction.

Species	Gene Fragment	Identifier	Prediction	Sequence length	relaxation	AF2 version	Figure
H. sapiens	HAUS2	NP_060567.1	N-clamp	1-118	N	2.2.0	1
	HAUS6	NP_060115.3	N-clamp	1-269	N	2.2.0	1
	HAUS7	NP_001372411.1	N-clamp	1-299	N	2.2.0	1
	HAUS8	NP_219485.1	N-clamp	1-248	N	2.2.0	1
X. laevis	HAUS2	NP_001085195.1	N-clamp	1-114	N	2.2.0	1
	HAUS6	NM_001097095.1	N-clamp	1-263	N	2.2.0	1
	HAUS7	NP_001121229.1	N-clamp	1-261	N	2.2.0	1
	HAUS8	NP_001090302.1	N-clamp	1-259	N	2.2.0	1
A. thaliana	AUG2	NP_565760.1	N-clamp	1-128	N	2.2.0	1
	AUG6	NP_568585.1	N-clamp	1-284	N	2.2.0	1
	AUG7	NP_568354.1	N-clamp	1-241	N	2.2.0	1
	AUG8	NP_001329921.1	N-clamp	1-533	N	2.2.0	1
D. melanogaster	Msd1	Q9W0G7	N-clamp	1-50	N	2.3.1	1
	Dgt6	Q9VAP2	N-clamp	1-280	N	2.3.1	1
	Msd5	Q9W0G6	N-clamp	1-179	N	2.3.1	1
	Dgt4	Q9W4M8	N-clamp	1-108	N	2.3.1	1
A. thaliana	AUG2	NP_565760.1	TII	1-296	N	2.2.0	Si 1
	AUG6	NP_568585.1	TII	1-741	N	2.2.0	Si 1
	AUG7	NP_568354.1	TII	1-329	N	2.2.0	Si 1
	AUG8	NP_001329921.1	TII	1-644	N	2.2.0	Si 1
D. melanogaster	Msd1	Q9W0G7	TII	1-138	N	2.3.1	Si 1
	Dgt6	Q9VAP2	TII	1-654	N	2.3.1	Si 1
	Msd5	Q9W0G6	TII	1-253	N	2.3.1	Si 1
	Dgt4	Q9W4M8	TII	1-188	N	2.3.1	Si 1
D. melanogaster	Dgt3	Q9W2P0	TIII	1 – 565	Y	2.3.2	Si 16
	Dgt5	Q7K4B4	TIII	1- 685	Y	2.3.2	Si 16
	Wac	Q9W0S8	TIII	1 – 163	Y	2.3.2	Si 16
	Dgt2	Q9VKD6	TIII	1 – 231	Y	2.3.2	Si 16
D. melanogaster	Dgt3	Q9W2P0	TII+Dgt3/5	88 – 393	Y	2.3.2	Si 16
	Dgt5	Q7K4B4	TII+Dgt3/5	85 – 530	Y	2.3.2	Si 16
	Msd1	Q9W0G7	TII+Dgt3/5	1-138	Y	2.3.2	Si 16
	Dgt6	Q9VAP2	TII+Dgt3/5	1-356	Y	2.3.2	Si 16
	Msd5	Q9W0G6	TII+Dgt3/5	1-253	Y	2.3.2	Si 16
	Dgt4	Q9W4M8	TII+Dgt3/5	1-188	Y	2.3.2	Si 16
H. sapiens	HAUS6	NP_060115.3	CH6-tubulin	1-156	Y	2.3.2	6
	TBA1A	P02550	CH6-tubulin	1-451	Y	2.3.2	6
	TUBBA	P02554	CH6-tubulin	1-445	Y	2.3.2	6
X. laevis	HAUS6	NM_001097095.1	CH6-tubulin	1-158	Y	2.3.2	6
	TBA1A	P02550	CH6-tubulin	1-451	Y	2.3.2	6
	TUBBA	P02554	CH6-tubulin	1-445	Y	2.3.2	6
A. thaliana	AUG6	NP_568585.1	CH6-tubulin	1-150	Y	2.3.2	6
	TBA1A	P02550	CH6-tubulin	1-451	Y	2.3.2	6
	TUBBA	P02554	CH6-tubulin	1-445	Y	2.3.2	6
D. melanogaster	Dgt6	Q9VAP2	CH6-tubulin	1-146	Y	2.3.2	6
	TBA1A	P02550	CH6-tubulin	1-451	Y	2.3.2	6
	TUBBA	P02554	CH6-tubulin	1-445	Y	2.3.2	6

Supplementary Table 8: Analysis of CH7 and HAUS8-NT Identifiers (UniProtKB) for HAUS7/Msd5 and HAUS8/Dgt4 sequences used in evolutionary comparison of HAUS7 and HAUS8 functional elements**.**

Organism	HAUS7/Msd5	HAUS8/Dgt4
D. melanogaster	Q9W0G6	Q9W4M8
D. busckii	A0A0M4EC86	UPI00083F3556
C. capitata	W8CCR4	W8C4H5
B. dorsalis	UPI0020C9AC0C	UPI0020C9E17B
R. pomonella	UPI0017854FE6	UPI00177D7193
T. dalmanni	UPI0018CD80B8	UPI0018CDDFD0
L. cuprina	A0A0L0BT39	A0A0L0C7U5
S. bullata	TMW51504.1	TMW54161.1
G. fuscipes	A0A9C5ZJ04	A0A8U0W9T1
M. domestica	A0A1I8MAP8	UPI0027BA3701
D. similis	UPI001EF85434	UPI001EF75908
O. abietinus	UPI00062594C5	UPI0006261165
A. gifuensis	UPI001CDC2A05	UPI001CDD6E76
L. heterotoma	UPI001CA832FF	UPI001CA94242
N. vitripennis	A0A7M7Q2N6	A0A7M7QLL2
T. cornetzi	A0A195DAI7	A0A195DAI7
H. saltator	E2BMJ0	E2BDM4
C. calcarata	A0AAJ7J521	A0AAJ7NA47
A. mellifera	A0A7M7L6C7	A0A7M7H172
B. bifarius	A0A6P8MAE2	A0A6P8N362
M. quadrifasciata	A0A0N0U5X0	A0A0M8ZX63
M. edulis	A0A8S3UT62	A0A8S3QK54
E. electricus	A0A4W4ELP7	A0A4W4FLL4
O. latipes	H2L9G5	A0A3B3I9P7
X. laevis	B1H1T5	Q0IHJ3
B. taurus	A6QQH2	A0AAA9SER2
M. musculus	Q8BKT8	Q99L00
H. sapiens	Q99871	Q9BT25
S. pistillata	A0A2B4SAN7	A0A2B4SVZ9
A. thaliana	A0A178UG29	Q9SUH5

Supplementary Table 9: Cryo-EM data collection, refinement and validation statistics

	D. melanogaster augmin N-clamp bound to a 14PF MT (EMDB-54174)	Helical reconstruction of a *D. melanogaster* augmin N-clamp-decorated 14PF MT (EMDB-52832)	*D. melanogaster* augmin GST-N-clamp bound to a 13PF MT (EMD-54160)	*D. melanogaster* augmin GST-N-clamp bound to a 13PF MT, well-defined subset of particles (EMDB-54161) (PDB 9RPD)
**Data collection and processing**				
Microscope	Titan Krios G1	Titan Krios G1	Titan Krios G1	Titan Krios G1
Camera	Gatan K3	Gatan K3	Gatan K3	Gatan K3
Magnification	81000	81000	81000	81000
Voltage (kV)	300	300	300	300
Electron exposure (e–/Å^2)	49.8	49.8	40.4	40.4
Defocus range (μm)	-1 to -3	-1 to -3	-1 to -3	-1 to -3
Pixel size (Å)	1.069	1.069	1.069	1.069
Symmetry imposed	C1	14-fold helical symmetry, twist -25.74°, rise 8.75 Å	C1	C1
Movies	8528	8528	8882	8882
Initial particle images (no.)	441250	441250	348721	348721
Final particle images (no.)	469266 (symmetry expanded)	102166	457096 (symmetry expanded)	117870 (symmetry expanded)
Map resolution (Å) FSC threshold	4.7 0.143	4.0 0.143	3.9 0.143	4.9 0.143
Map resolution range (Å)	3.0 -10.0	3.8 – 9.0	4.3 - 7.8	3.3 - 7.1
**Refinement**				
Initial model used	-	-	-	AF2 model (Supplementary Figure 1; Supplementary Table 7)
Model resolution (Å) FSC threshold	-	-	-	11 (FSC threshold 0.5); 4.9 (FSC threshold 0.143)
Model resolution range (Å)	-	-	-	-
Map sharpening *B* factor (Å^2)	-62	-149	-25	-21
**Model composition**	-	-	-	
Non-hydrogen atoms				41510
Protein residues				2772
Ligands				-
***B* factors (Å^2)**	-	-	-	
Protein				92.57
Ligand				-
**R.m.s. deviations**	-	-	-	
Bond lengths (Å)				0.013
Bond angles (°)				1.704
**Validation**	-	-	-	
MolProbity score				1.9
Clashscore				16
Poor rotamers (%)				1
**Ramachandran plot**	-	-	-	
Favored (%)				97
Allowed (%)				3
Disallowed (%)				0

References

1. Katoh, K., Misawa, K., Kuma, K. & Miyata, T. MAFFT: a novel method for rapid multiple sequence alignment based on fast Fourier transform. *Nucleic Acids Res* **30**, 3059–3066 (2002).
2. Waterhouse, A. M., Procter, J. B., Martin, D. M. A., Clamp, M. & Barton, G. J. Jalview Version 2—a multiple sequence alignment editor and analysis workbench. *Bioinformatics* **25**, 1189–1191 (2009).
3. Cook, A. D., Manka, S. W., Wang, S., Moores, C. A. & Atherton, J. A microtubule RELION-based pipeline for cryo-EM image processing. *J Struct Biol* **209**, 107402 (2020).
4. Asarnow Daniel; Palovcak, E. C. Y. asarnow/pyem: UCSF pyem v0.5. Preprint at (2019).
5. Elfmann, C. & Stülke, J. PAE viewer: a webserver for the interactive visualization of the predicted aligned error for multimer structure predictions and crosslinks. *Nucleic Acids Res* **51**, W404–W410 (2023).
6. Gao, Q. *et al.* Structural mechanisms for centrosomal recruitment and organization of the microtubule nucleator γ-TuRC. *Nat Commun* **16**, 2453 (2025).
