# Supplementary material for: Conserved function of the HAUS6 calponin homology domain in anchoring augmin for microtubule branching
Source: Nat Commun. 2025 Aug 22;16:7845. doi: 10.1038/s41467-025-63165-z (PMC12373997; doi:10.1038/s41467-025-63165-z)
Supplement: Supplementary file 2 — Description of Additional Supplementary Files [file 41467_2025_63165_MOESM2_ESM.pdf]

Description of Additional Supplementary Files

File name: Supplementary Movie 1
Description: MD simulation of MT-bound *D. melanogaster* augmin N-clamp: Shown is augmin N-clamp and one row of β-tubulin molecules. Coloring as in Fig. 5d.
