# Supplementary material for: Conserved function of the HAUS6 calponin homology domain in anchoring augmin for microtubule branching
Source: Nat Commun. 2025 Aug 22;16:7845. doi: 10.1038/s41467-025-63165-z (PMC12373997; doi:10.1038/s41467-025-63165-z)
Supplement: Supplementary file 4 — Reporting Summary [file 41467_2025_63165_MOESM4_ESM.pdf]

nature portfolio

Corresponding author(s): Martin Würtz, Stefan Pfeffer

Last updated by author(s): 2025/8/3

Reporting Summary

Nature Portfolio wishes to improve the reproducibility of the work that we publish. This form provides structure for consistency and transparency in reporting. For further information on Nature Portfolio policies, see our Editorial Policies and the Editorial Policy Checklist.

Statistics

For all statistical analyses, confirm that the following items are present in the figure legend, table legend, main text, or Methods section.

n/a	Confirmed	
☐	☒	The exact sample size (n) for each experimental group/condition, given as a discrete number and unit of measurement
☐	☒	A statement on whether measurements were taken from distinct samples or whether the same sample was measured repeatedly
☐	☒	The statistical test(s) used AND whether they are one- or two-sided<br>*Only common tests should be described solely by name; describe more complex techniques in the Methods section.*
☒	☐	A description of all covariates tested
☒	☐	A description of any assumptions or corrections, such as tests of normality and adjustment for multiple comparisons
☐	☒	A full description of the statistical parameters including central tendency (e.g. means) or other basic estimates (e.g. regression coefficient) AND variation (e.g. standard deviation) or associated estimates of uncertainty (e.g. confidence intervals)
☐	☒	For null hypothesis testing, the test statistic (e.g. F, t, r) with confidence intervals, effect sizes, degrees of freedom and P value noted<br>*Give P values as exact values whenever suitable.*
☒	☐	For Bayesian analysis, information on the choice of priors and Markov chain Monte Carlo settings
☒	☐	For hierarchical and complex designs, identification of the appropriate level for tests and full reporting of outcomes
☐	☒	Estimates of effect sizes (e.g. Cohen's d, Pearson's r), indicating how they were calculated

Our web collection on statistics for biologists contains articles on many of the points above.

Software and code

Policy information about availability of computer code

Data collection	Cryo-EM data was collected using EPU v. 2.6, SPA, on a Krios TEM equipped with a Gatan K3 camera operated by Gatan Microscopy Suite v. 3.32. Representative images collected during screening of cryo-EM samples and indicated in the manuscript were collected using EPU on a Glacios TEM.<br>Negative stain EM data were acquired on a Talos L120C TEM (Thermo Fisher Scientific) operated at 120 kV, room temperature, and equipped with a 4k x 4k Ceta CMOS camera (Thermo Fisher Scientific).<br>Data for immunoblots was collected using LAS4000 v. 2.1. Immunofluorescence acquisition was performed using a DeltaVision RT system (Applied Precision) with an Olympus IX71 microscope, equipped with 60×/1.42 and 100×/1.40 oil objective lenses.<br>qPCR was performed in triplicates on a LightCycler 480 multiwell plate, using LightCycler 480 SYBR Green I Master (Roche) in combination with the LightCycler 480 instrument (Roche).<br>Chromatography runs were performed using an ÄktaPure instrument (Cytiva). Mass photometry was measured using a Refeyn TwoMP mass photometer (Refeyn Ltd, Oxford, UK). Nanoflow LC-MS/MS analysis was performed using an Ultimate 3000 liquid chromatography system coupled to an Orbitrap QE HF (Thermo Fisher Scientific) or the Vanquish Neo coupled to an Orbitrap Tribrid Eclipse (Thermo Fisher Scientific).
Data analysis	EM data were processed using crYOLO v. 1.8, Relion v. 3.0, 3.1 and 5.0, MotionCor2 v. 1.0.5, Gctf v. 1.06, CryoSPARC v. 4.4.1 and v. 4.5.3, MiRP v. 2 and pyem. All atomic model and density-related analysis and visualization was done using UCSF Chimera v. 1.14 and v. 1.16 and UCSF ChimeraX v. 1.3, 1.4, 1.7.1 and 1.9. AlphaFold predictions were carried out using AlphaFold Multimer v. 2.2.0, 2.3.1 and 2.3.2. Plots of the Predicted Aligned Error were generated using the PAE viewer tool.The atom renumbering of the composite octamer structural model was carried out with pdb-tools. Conservation scores for the CH domains were estimated using the ConSurf server.<br>Generation of plots and statistical analysis was done with Graphpad PRISM v. 10.0. Immunofluorescent data was analyzed using Fiji software v2.14.0. The Äkta pure and Äkta go systems were controlled by Unicorn software v.7.5 or 7.9. Mass photometry data were analyzed using Refeyn DiscoverMP 2024 R1 software (Refeyn Ltd, Oxford, UK). MD data were analysed using MDAnalysis and VMD.The parameters for GDP

and GTP molecules were generated using CHARMM-GUI v1.7, specifically through the Ligand Reader & Modeler tool. MS data generated with Orbitrap QE HF were searched using Proteome Discoverer v. 2.5 with Sequest HT (Thermo Fisher Scientific). Data generated with Orbitrap Tribrid Eclipse were searched using Proteome Discoverer v. 3.1 with Sequest HT (Thermo Fisher Scientific). The MS/MS spectra were searched against the following databases: the customized contaminant database (part of MaxQuant, MPI Martinsried).
Fitting and kinetic parameter calculations were performed using least squares fitting with the SciPy Python library , and data were plotted using Matplotlib.MSA was generated with the MAFFT algorithm using standard parameters and visualized in JalView

For manuscripts utilizing custom algorithms or software that are central to the research but not yet described in published literature, software must be made available to editors and reviewers. We strongly encourage code deposition in a community repository (e.g. GitHub). See the Nature Portfolio guidelines for submitting code & software for further information.

Data

Policy information about availability of data

All manuscripts must include a data availability statement. This statement should provide the following information, where applicable:

- Accession codes, unique identifiers, or web links for publicly available datasets
- A description of any restrictions on data availability
- For clinical datasets or third party data, please ensure that the statement adheres to our policy

Atomic coordinates and cryo-EM densities have been deposited at the Protein Data Bank and the Electron Microscopy Data Bank under accession codes: PDB 9RPD [https://www.rcsb.org/structure/9RPD] and EMD-54161 [https://www.ebi.ac.uk/emdb/EMD-54161] (D. melanogaster augmin GST-N-clamp bound to a MT, well-defined subset of particles), EMD-54174 [https://www.ebi.ac.uk/emdb/EMD-54174] (D. melanogaster augmin N-clamp bound to a MT), EMD-52832 [https://www.ebi.ac.uk/emdb/EMD-52832] (helical reconstruction of a D. melanogaster N-clamp-decorated MT), EMD-54160 [https://www.ebi.ac.uk/emdb/EMD-54160] (D. melanogaster augmin GST-N-clamp bound to a MT). Published structural data used in this article are: PDB 7SQK [https://www.rcsb.org/structure/7SQK], PDB 6WVR [https://www.rcsb.org/structure/6WVR], PDB 3IZ0 [https://www.rcsb.org/structure/3IZ0], PDB 7PT5 [https://www.rcsb.org/structure/7PT5] and PDB 8AT3 [https://www.rcsb.org/structure/8AT3]. The models predicted by AF2 generated in this study have been deposited in the ModelArchive database with the identifiers ma-vxlav [https://modelarchive.org/doi/10.5452/ma-vxlav] (X. laevis augmin N-clamp), ma-apjck [https://modelarchive.org/doi/10.5452/ma-apjck] (human augmin N-clamp), ma-a7krk [https://modelarchive.org/doi/10.5452/ma-a7krk] (D. melanogaster augmin N-clamp), ma-u3pr2 [https://modelarchive.org/doi/10.5452/ma-u3pr2] (A. thaliana augmin N-clamp), ma-t3gx3 [https://modelarchive.org/doi/10.5452/ma-t3gx3] (A. thaliana augmin TII), ma-5p38z [https://modelarchive.org/doi/10.5452/ma-5p38z] (D. melanogaster augmin TII), ma-8pbts [https://modelarchive.org/doi/10.5452/ma-8pbts] (D. melanogaster augmin TIII), ma-7w5lz [https://modelarchive.org/doi/10.5452/ma-7w5lz] (D. melanogaster augmin TII+III(interface)), ma-3drto [https://modelarchive.org/doi/10.5452/ma-3drto] (D. melanogaster augmin CH6 with tubulin dimer), ma-5rtzo [https://modelarchive.org/doi/10.5452/ma-5rtzo] (human augmin CH6 with tubulin dimer), ma-xcax9 [https://modelarchive.org/doi/10.5452/ma-xcax9] (A. thaliana augmin CH6 with tubulin dimer), ma-upjzh [https://modelarchive.org/doi/10.5452/ma-upjzh] (X. laevis augmin CH6 with tubulin dimer). Mass spectrometry data of purified augmin N-clamp complexes are available from the ProteomeXchange Consortium 108 via the PRIDE partner repository 109 with the dataset identifier PXD060099 [https://proteomecentral.proteomexchange.org/cgi/GetDataset?ID= PXD060099]. The initial configuration files for the Molecular Dynamics simulations associated with this study and the trajectories have been deposited on ZENODO. They are publicly available at the following address: https://doi.org/10.5281/zenodo.16452427110. The script used for quantitative analysis and plotting of the tubulin co-sedimentation assays is available at https://github.com/gt-biomodel/tubulin-co-sedimentation-plots and at https://doi.org/10.5281/zenodo.16452427110. Supplementary Information and Source Data are provided with this paper.

Research involving human participants, their data, or biological material

Policy information about studies with human participants or human data. See also policy information about sex, gender (identity/presentation), and sexual orientation and race, ethnicity and racism.

Reporting on sex and gender	*Use the terms sex (biological attribute) and gender (shaped by social and cultural circumstances) carefully in order to avoid confusing both terms. Indicate if findings apply to only one sex or gender; describe whether sex and gender were considered in study design; whether sex and/or gender was determined based on self-reporting or assigned and methods used. Provide in the source data disaggregated sex and gender data, where this information has been collected, and if consent has been obtained for sharing of individual-level data; provide overall numbers in this Reporting Summary. Please state if this information has not been collected. Report sex- and gender-based analyses where performed, justify reasons for lack of sex- and gender-based analysis.*
Reporting on race, ethnicity, or other socially relevant groupings	*Please specify the socially constructed or socially relevant categorization variable(s) used in your manuscript and explain why they were used. Please note that such variables should not be used as proxies for other socially constructed/relevant variables (for example, race or ethnicity should not be used as a proxy for socioeconomic status). Provide clear definitions of the relevant terms used, how they were provided (by the participants/respondents, the researchers, or third parties), and the method(s) used to classify people into the different categories (e.g. self-report, census or administrative data, social media data, etc.) Please provide details about how you controlled for confounding variables in your analyses.*
Population characteristics	*Describe the covariate-relevant population characteristics of the human research participants (e.g. age, genotypic information, past and current diagnosis and treatment categories). If you filled out the behavioural & social sciences study design questions and have nothing to add here, write "See above."*
Recruitment	*Describe how participants were recruited. Outline any potential self-selection bias or other biases that may be present and how these are likely to impact results.*
Ethics oversight	*Identify the organization(s) that approved the study protocol.*

Note that full information on the approval of the study protocol must also be provided in the manuscript.

Field-specific reporting

Please select the one below that is the best fit for your research. If you are not sure, read the appropriate sections before making your selection.

[x] Life sciences [ ] Behavioural & social sciences [ ] Ecological, evolutionary & environmental sciences

For a reference copy of the document with all sections, see nature.com/documents/nr-reporting-summary-flat.pdf

Life sciences study design

All studies must disclose on these points even when the disclosure is negative.

Sample size	For EM data, no statistical method was chosen to determine the sample size. The number of micrographs was chosen to obtain a number of particles sufficient to reconstruct a 3D density with the stated resolution determined with Gold Standard FSC method. AlphaFold predictions produced 5x5 (AlphaFold2) models per construct. For immunofluorescent experiments, images were acquired in number that is sufficient for statistical analysis (as specified in figure legend and Methods). For Tubulin co-sedimentation assays data points were choosed to have suffiecent data points to fit the data.
Data exclusions	For cryo-EM SPA and negative stain EM, all EM images were used for particle picking. Cryo-EM particle selection was performed after 3D classifcation sorting for protofilaments. Afterwards processing was done either with13 (GST-N-clamp) 14PF (wild-type N-clamp) as it were the classes with most particles. After routine pipline all particles were used for refinments and then sorted in 3D classifcation and the quality of resulting 3D maps was the exclusion criterion, as is standard image processing. For the immunofluorescence data, all cells in metaphase were analyzed. For tubulin co-sedimentation assays all samples that did not pellet in control condition were used for analysis. Only data points with the same concentrations were used for comparision between differnt samples.
Replication	Cryo-EM SPA and negative stain EM data were acquired on one grid. 3D densities are averages of hundreds of thousands of particles and thus repetitions of the experiments were not necessary. Immunofluorescence and immunoblot experiments were repeated 3 times (unless specified in figure legend and Methods). Mass photometry measurements were done once, as replica were not required to asses the molecular mass of the prufied complexes. Tubulin co-sedimentation assays were perfomed in at least 3 replicates. Protein expressions and purifcations were repeated at least 3 times. All experiments that were repeated could be replicated.
Randomization	Positions for image acquisition in cryo-EM and negative stain EM were selected based on the presence of the particles. In immunofluorescent experiments for each condition metaphase cells were aquired randomly on the slides and only metaphase cells were analysed. Other experiments were not related to randomization.
Blinding	AF2 and EM analysis was not blinded because it was performed computationally. For immunofluorescence experiments it was not feasible to apply blinding experiments because of large sample sizes. Generally the results from experiments were confirmed by independent approaches e.g. immunofluorescence and tubulin co-sedimentation assays experiments. For other experiments like cloning, expression protein purification it was technically not possible.

Reporting for specific materials, systems and methods

We require information from authors about some types of materials, experimental systems and methods used in many studies. Here, indicate whether each material, system or method listed is relevant to your study. If you are not sure if a list item applies to your research, read the appropriate section before selecting a response.

Materials & experimental systems

n/a	Involved in the study
[ ]	[x] Antibodies
[ ]	[x] Eukaryotic cell lines
[x]	[ ] Palaeontology and archaeology
[x]	[ ] Animals and other organisms
[x]	[ ] Clinical data
[x]	[ ] Dual use research of concern
[x]	[ ] Plants

Methods

n/a	Involved in the study
[x]	[ ] ChIP-seq
[x]	[ ] Flow cytometry
[x]	[ ] MRI-based neuroimaging

Antibodies

Antibodies used	Primary antibodies used in this study for immunofluorescence (IF) and immunoblot were: GFP was stained using A10262 chicken antibody (1:200, Thermo Fisher). In D. melanogaster S2 cells α-tubulin was stained with Sigma T9026 mouse antibody (1:700), whereas for RPE1 cells mouse α-tubulin (monoclonal, clone 1E4C11, Proteintech, IF 1:500, REF 66031-1IG, LOT 10028345) was used. Alpaca HRP-conjugated sdAb anti-ALFA (monoclonal, clone 1G5, NanoTag Biotechnologies GmbH, WB 1:1000, REF N1505-HRP, LOT 032303B), rabbit GAPDH (monoclonal, clone 14C10F5G9, Cell Signaling Technology, WB 1:1000, REF #2118, LOT 16), rabbit polyclonal antibodies against HAUS6 (polyclonal, AA 448–955, WB 1:2000, IF 1:1000) 72.<br>Secondary HRP conjugated antibody used in immunoblot was HRP-conjugated anti-rabbit (polyclonal, Jackson ImmunoResearch, WB 1:5000, REF 711-035-152, 172401). Secondary antibodies used for immunofluorescence were: Alexa Fluor 488 goat anti-chicken

(Thermo Fisher Scientific, A-11039, 1:500), Alexa Fluor 647 goat anti-mouse (Thermo Fisher Scientific, A-21235, 1:500), Alexa Fluor Plus 488 donkey anti-mouse IgG (polyclonal, Thermo Fisher Scientific, IF 1:500, REF A-11001, LOT 2816171), Alexa Fluor Plus 647 donkey anti-rabbit IgG (polyclonal, Thermo Fisher Scientific, IF 1:500, REF A32733, LOT 2577247).

Validation	(Thermo Fisher, A10262: https://www.scbt.com/p/gfp-antibody-c-2?gad_source=1&gbraid=0AAAAAD8VVLfxetOf67OVbiS6AEQK01VB1&gclid=Cj0KCQiAwOe8BhCCARIsAGKeD54EKRaUzMfj1JWSnL5Zgs9eUAj3i0oVDX12dmCQV8QtwoR9u93tG3caAkofEALw_wcB)<br><br>(Sigma, T9026: https://www.sigmaaldrich.com/DE/de/product/sigma/t9026?utm_source=google&utm_medium=cpc&utm_id=12410876063&utm_campaign=%7Bcampaignname%7D&utm_content=120745830949&utm_term=sigma+t9026&gad_source=1&gbraid=0AAAAAD8kLQQzw_FTecqCagWFWVYjPjFjo&gclid=Cj0KCQiAwOe8BhCCARIsAGKeD573t1gJUpib-J4EnyiETiTyf8f6yjK6TBydNUJ_wFiQ1kbjYLn9tn8aApVWEALw_wcB)<br><br>(Proteintech, 11224-1-AP: https://www.ptglab.com/products/Pictures/pdf/11224-1-AP.pdf?srsltid=AfmBOoqwd-H2zfLlwdzF5V9B0qnef1kQAo1XPQEJHZJ5-PHn8EY3g8oi)<br><br>(NanoTag Biotechnologies GmbH, N1505-HRP: https://nano-tag.com/product/anti-alfa-sdab/)<br><br>(ell Signaling Technology, 14C10: https://www.cellsignal.com/products/primary-antibodies/gapdh-14c10-rabbit-mab/2118?srsltid=AfmBOopuY2B74NuXj2Gmzjmao0kseNdg721jf6rHjkEWUm-tmTO1YJkg)<br><br>(rabbit polyclonal antibodies against HAUS6: https://doi.org/10.1038/ncb2996)<br><br>(Jackson, 711-035-152: https://www.jacksonimmuno.com/catalog/products/711-035-152)<br><br>(Thermo Fisher Scientific, A-1103 9https://www.thermofisher.com/antibody/product/Goat-anti-Chicken-IgY-H-L-Secondary-Antibody-Polyclonal/A-11039)<br><br>(Thermo Fisher Scientific, A-21235, 1:500 hhttps://www.thermofisher.com/antibody/product/Goat-anti-Mouse-IgG-H-L-Cross-Adsorbed-Secondary-Antibody-Polyclonal/A-21235)<br><br>(Thermo Fisher Scientific, A-11001: https://www.thermofisher.com/antibody/product/Goat-anti-Mouse-IgG-H-L-Cross-Adsorbed-Secondary-Antibody-Polyclonal/A-11001)<br><br>(Thermo Fisher Scientific, A32733: https://www.thermofisher.com/antibody/product/Goat-anti-Rabbit-IgG-H-L-Highly-Cross-Adsorbed-Secondary-Antibody-Polyclonal/A32733)

Eukaryotic cell lines

Policy information about cell lines and Sex and Gender in Research

Cell line source(s)	RPE1 cell line are as described (https://doi.org/10.1038/s41467-020-14767-2).<br>Drosophila melanogaster S2 cells were a gift of the Boutros Lab DKFZ, Heidelberg.
Authentication	We verified all cell lines according to the morphology by light microscopy.
Mycoplasma contamination	RPE1 cells were tested and were negative in the mycoplasma contamination test. Drosophila melanogaster S2 cells were not tested
Commonly misidentified lines<br>(See ICLAC register)	No commonly misidentified lines were used in this study.

Plants

Seed stocks

Report on the source of all seed stocks or other plant material used. If applicable, state the seed stock centre and catalogue number. If plant specimens were collected from the field, describe the collection location, date and sampling procedures.

Novel plant genotypes

Describe the methods by which all novel plant genotypes were produced. This includes those generated by transgenic approaches, gene editing, chemical/radiation-based mutagenesis and hybridization. For transgenic lines, describe the transformation method, the number of independent lines analyzed and the generation upon which experiments were performed. For gene-edited lines, describe the editor used, the endogenous sequence targeted for editing, the targeting guide RNA sequence (if applicable) and how the editor was applied.

Authentication

Describe any authentication procedures for each seed stock used or novel genotype generated. Describe any experiments used to assess the effect of a mutation and, where applicable, how potential secondary effects (e.g. second site T-DNA insertions, mosiacism, off-target gene editing) were examined.
